# A Mutation in *LTBP2* Causes Congenital Glaucoma in Domestic Cats (*Felis catus*)

**DOI:** 10.1371/journal.pone.0154412

**Published:** 2016-05-05

**Authors:** Markus H. Kuehn, Koren A. Lipsett, Marilyn Menotti-Raymond, S. Scott Whitmore, Todd E. Scheetz, Victor A. David, Stephen J. O'Brien, Zhongyuan Zhao, Jackie K. Jens, Elizabeth M. Snella, N. Matthew Ellinwood, Gillian J. McLellan

**Affiliations:** 1 Department of Ophthalmology and Visual Sciences, Carver College of Medicine, University of Iowa, Iowa City, Iowa, United States of America; 2 Laboratory of Genomic Diversity, National Cancer Institute, Frederick, Maryland, United States of America; 3 Basic Research Laboratory, National Cancer Institute, Frederick, Maryland, United States of America; 4 Department of Chemistry, Gettysburg College, Gettysburg, Pennsylvania, United States of America; 5 Theodosius Dobzhansky Center for Genome Bioinformatics, St. Petersburg State University, St. Petersburg, Russia; 6 Oceanographic Center, Nova Southeastern University, Fort Lauderdale, Florida, United States of America; 7 Department of Animal Science, Iowa State University, Ames, Iowa, United States of America; 8 Department of Veterinary Clinical Sciences, Iowa State University, Ames, Iowa, United States of America; 9 Department of Ophthalmology and Visual Sciences, University of Wisconsin-Madison, Madison, Wisconsin, United States of America; 10 Department of Surgical Sciences, University of Wisconsin-Madison, Madison, Wisconsin, United States of America; 11 McPherson Eye Research Institute, Madison, Wisconsin, United States of America; Hanson Institute, AUSTRALIA

## Abstract

The glaucomas are a group of diseases characterized by optic nerve damage that together represent a leading cause of blindness in the human population and in domestic animals. Here we report a mutation in *LTBP2* that causes primary congenital glaucoma (PCG) in domestic cats. We identified a spontaneous form of PCG in cats and established a breeding colony segregating for PCG consistent with fully penetrant, autosomal recessive inheritance of the trait. Elevated intraocular pressure, globe enlargement and elongated ciliary processes were consistently observed in all affected cats by 8 weeks of age. Varying degrees of optic nerve damage resulted by 6 months of age. Although subtle lens zonular instability was a common feature in this cohort, pronounced ectopia lentis was identified in less than 10% of cats examined. Thus, glaucoma in this pedigree is attributed to histologically confirmed arrest in the early post-natal development of the aqueous humor outflow pathways in the anterior segment of the eyes of affected animals. Using a candidate gene approach, significant linkage was established on cat chromosome B3 (LOD 18.38, θ = 0.00) using tightly linked short tandem repeat (STR) loci to the candidate gene, *LTBP2*. A 4 base-pair insertion was identified in exon 8 of *LTBP2* in affected individuals that generates a frame shift that completely alters the downstream open reading frame and eliminates functional domains. Thus, we describe the first spontaneous and highly penetrant non-rodent model of PCG identifying a valuable animal model for primary glaucoma that closely resembles the human disease, providing valuable insights into mechanisms underlying the disease and a valuable animal model for testing therapies.

## Introduction

Glaucoma is a leading cause of blindness due to characteristic damage to the retinal ganglion cells (RGCs) and optic nerve, and in the human population is estimated to affect more than 60 million people worldwide [1]. Maintenance of an intraocular pressure (IOP) within a defined normal range is vital to the structural and physiological well-being of the eye. Aqueous humor is continuously produced by the ciliary body epithelium and is drained from the anterior chamber of the eye to the systemic circulation, primarily through the trabecular meshwork and Schlemm’s canal located just anterior to the root of the iris. The IOP is the result of the dynamic relationship between aqueous humor production and outflow. Obstruction of aqueous humor outflow leads to elevated IOP, which is an important risk factor for the development of glaucoma [2].

Although glaucoma is generally considered a disease of older adults, it is an important and devastating cause of vision loss in children, accounting for just under 7% of cases of childhood blindness worldwide [3–5]. A number of chromosomal loci associated with primary congenital glaucoma (PCG) have been identified in humans (OMIM 231300, 600975, 613085, and 613086) and mutations in the cytochrome P450 gene, *CYP1B1*, and *LTBP2* gene at two of these loci were identified in families segregating PCG [6, 7].

However, despite advances in our knowledge of glaucoma genetics, basic research in the pathogenesis and treatment of glaucoma in general, and PCG in particular, has been hampered by a lack of appropriate animal models [8]. Spontaneous glaucoma has been reported in a number of species including dogs and cats [9, 10] and an underlying molecular genetic basis reported for open angle glaucoma in Beagles [11]and Norwegian elkhounds [12] and angle closure glaucoma in Basset hounds [13]. We have established and characterized a novel, spontaneous, feline model of PCG. The phenotype was initially observed in a pair of Siamese cats but, as reported previously and demonstrated here, is independent of the Siamese genetic background [14]. Here we report the causal mutation in *LTBP2*, a known gene implicated in human PCG (OMIM 613086) [7, 15]. This represents the first extant, large animal model of PCG and is the first report documenting a causal mutation for glaucoma in cats.

## Results

### Clinical phenotype of feline PCG

**Fig 1:**
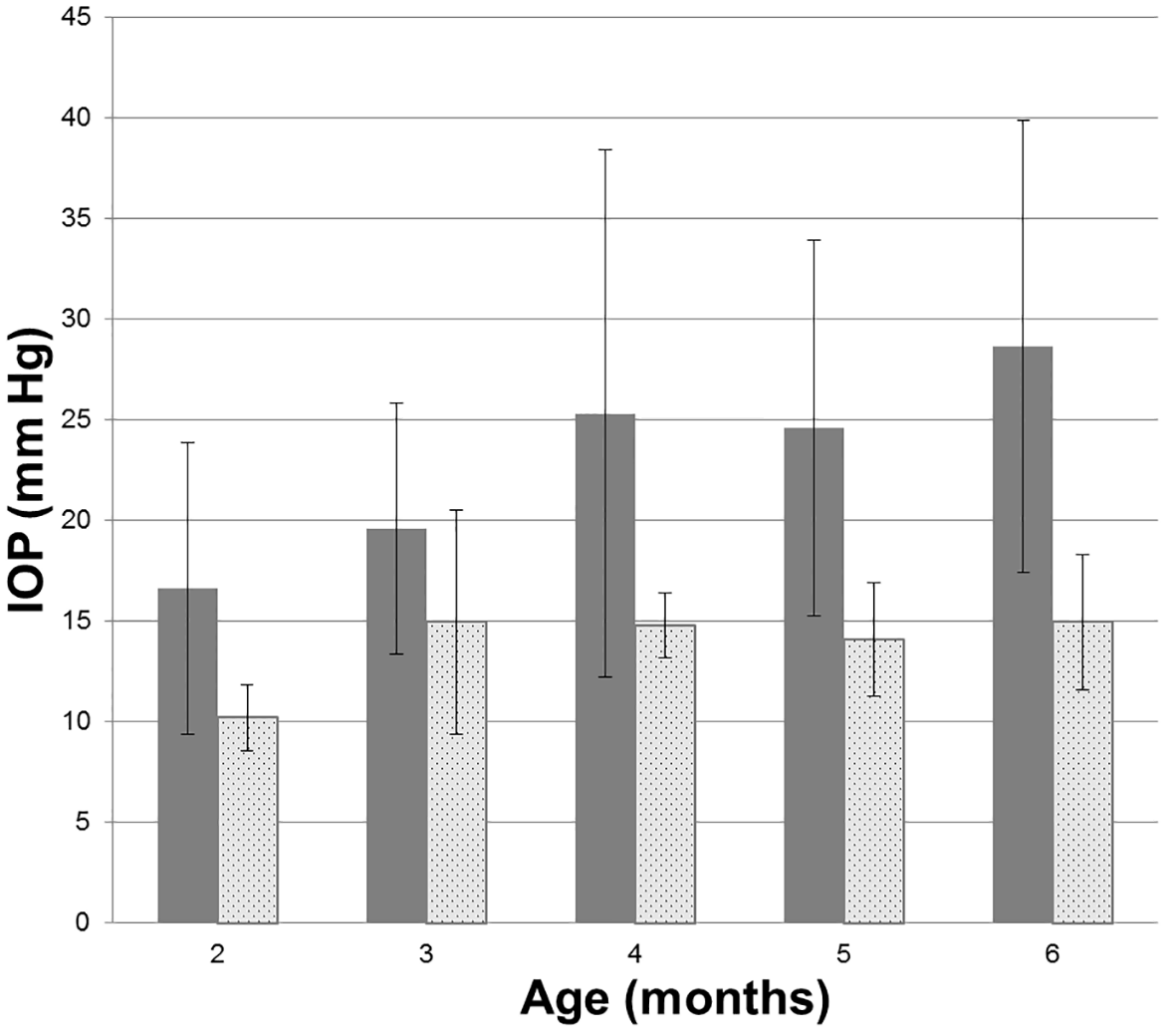
Intraocular pressure in glaucomatous and normal cats. Intraocular pressure measured by rebound tonometry (TonoVet, ICare oy, Finland) was significantly greater in PCG (solid bars) than in normal cats (patterned bars) at all ages from 2 months of age (ANOVA with Tukey-Kramer multiple comparisons post-test, p<0.05). Error bars represent standard deviation. Numbers (n) of subjects in each age group are indicated on each column.

Clinical examination to determine ocular phenotype was conducted in 147 cats in an expanded pedigree. Apart from the ocular phenotypes, the proband siblings and the two founding individuals appeared to be healthy, with no overt systemic abnormalities and were fertile. Neonatal mortality and incidence of other congenital abnormalities, such as cleft palate or cardiac defects was comparable to, or lower than, reported for purebred cats in other establishments and the general feline population. A distinctive, abnormal ocular phenotype was identified in all offspring that resulted from matings between these two Siamese individuals, and from other affected to affected pairings in the pedigree. All affected cats have bilateral mild to moderate globe enlargement that is generally apparent as soon as eye examinations can be reliably carried out in kittens. The disease is typically bilateral and symmetrical and no significant differences between the left and right eyes were observed. By the age of 8 weeks mean IOP is significantly higher in cats with congenital glaucoma than in normal cats (Fig 1). With the exception of <2% of animals examined that had grossly enlarged globes and corneal edema, all affected cats were subjectively assessed to be visual. Neither episcleral vascular congestion nor signs suggestive of ocular pain were apparent or attributable to the glaucoma phenotype and there was no evidence of intraocular inflammation on slit-lamp biomicrosopy.

**Fig 2:**
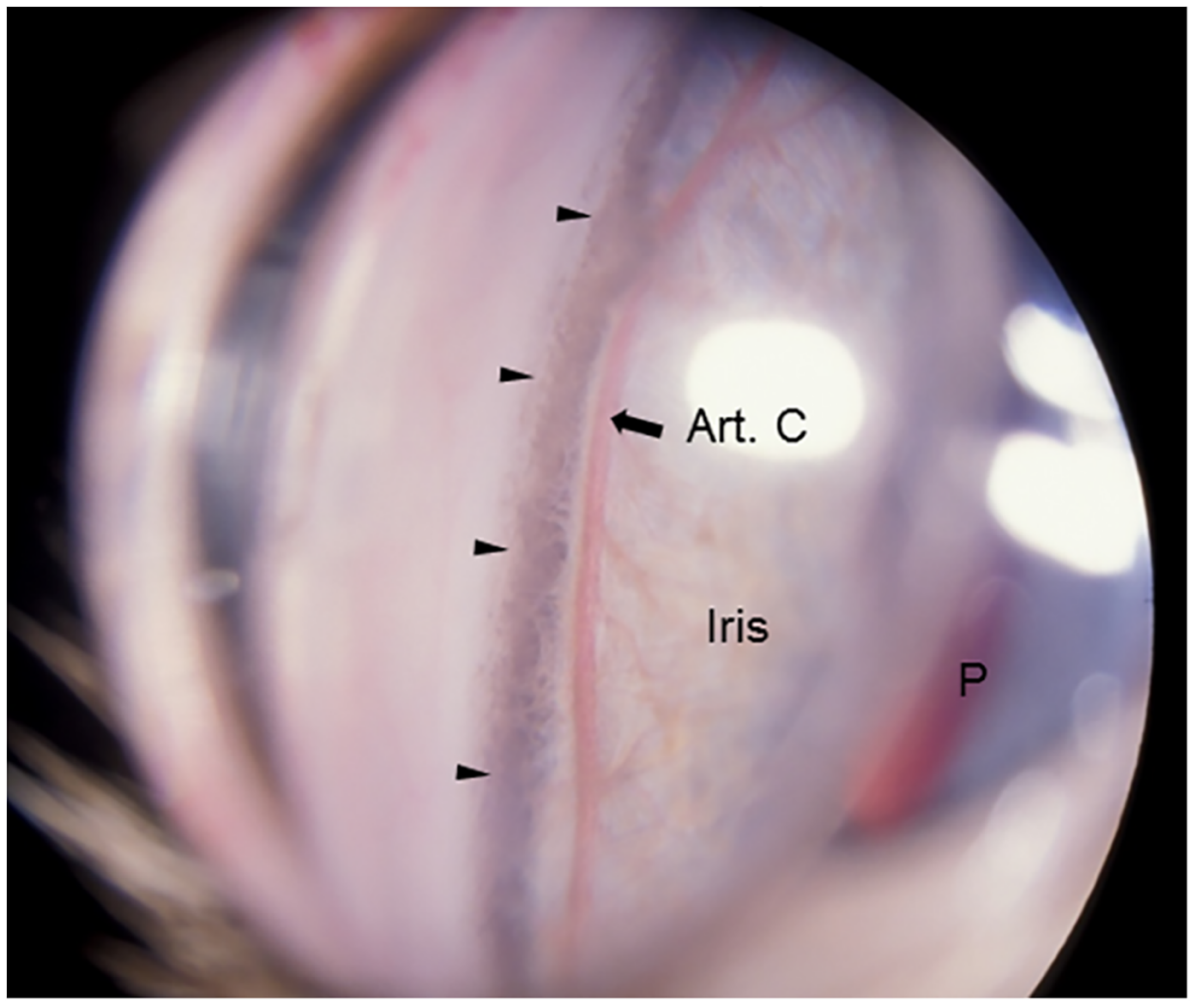
Gonioscopic Features of Feline Primary Congenital Glaucoma. Goniophotograph illustrates an open, but slightly narrowed, iridocorneal angle (opening of ciliary cleft; indicated by arrow heads) and mild dysplasia of the pectinate ligament visualized by gonioscopy in a 7 month-old Siamese cat with primary congenital glaucoma. No ectopia lentis was evident in this subject. (I = Iris, P = pupil, Art. C = major arterial circle of iris).

**Fig 3:**
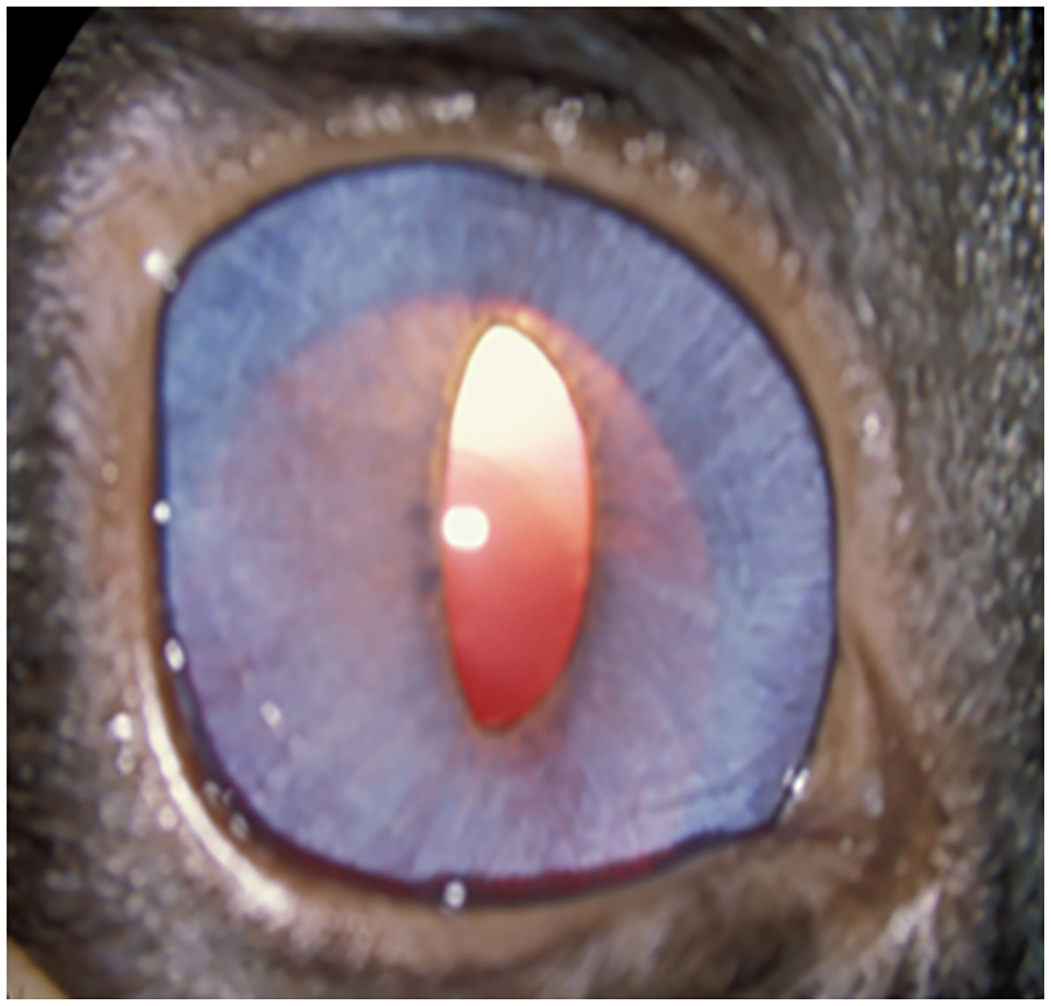
Clinical Appearance of Feline Primary Congenital Glaucoma. Iris hypoplasia and ectopia lentis in feline PCG. The outline of the posteriorly subluxated lens can be readily appreciated through thin, translucent iris tissue in this affected Siamese cat.

**Fig 4:**
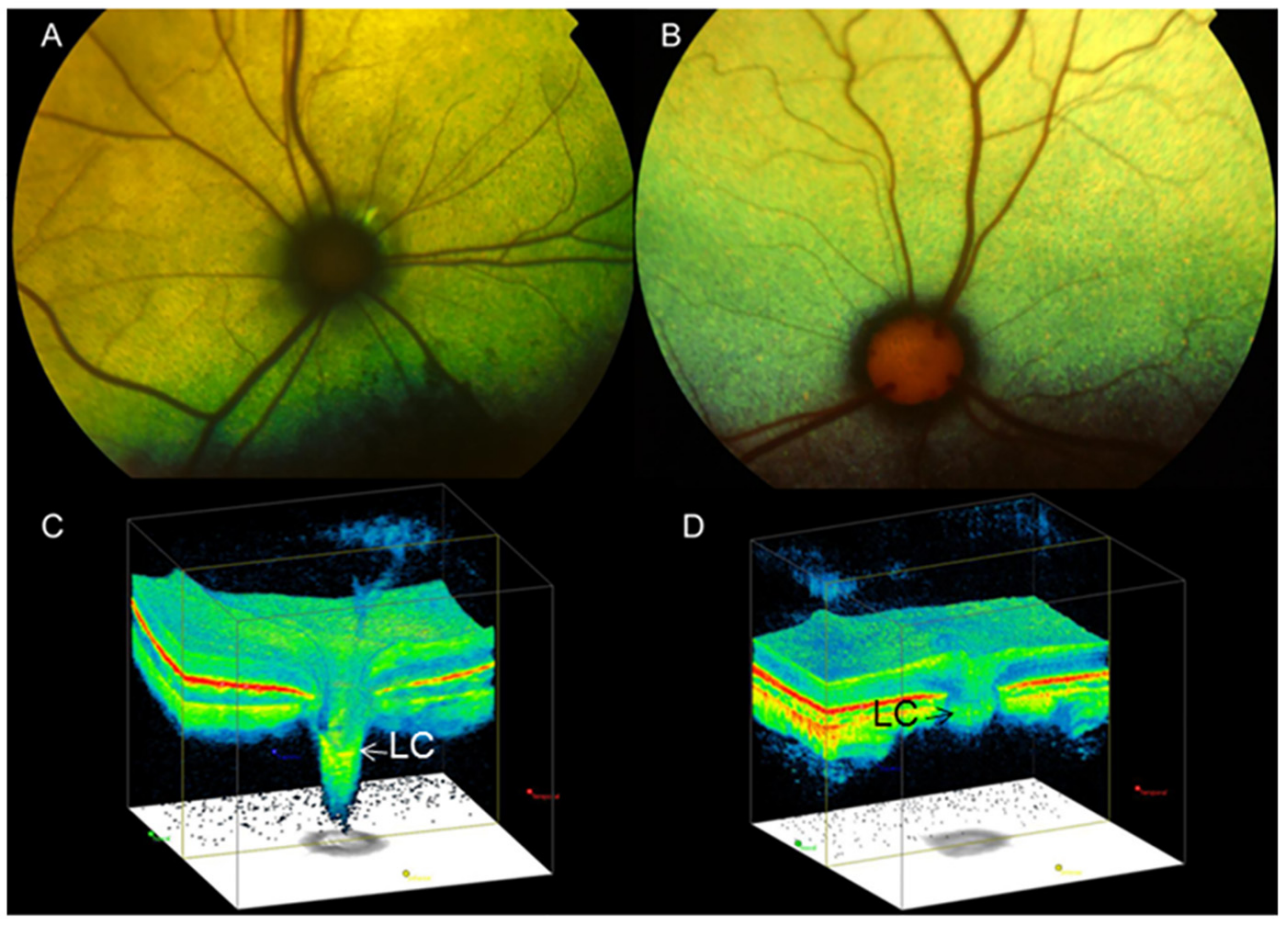
Optic Nerve Head Cupping in Feline Primary Congenital Glaucoma. Fundus photographs illustrate the cupped and degenerate optic nerve head of [A] a cat with advanced primary congenital glaucoma and compared with a normal age-matched cat [B]. An optic nerve cube scan obtained by spectral domain optical coherence tomography (OCT;Cirrus, Carl Zeiss Meditec Inc., Dublin, CA) acquired in this affected cat confirms dramatic posterior displacement of the lamina cribrosa (LC, arrowed)[C] compared to the normal control[D]. (Modified, with permission, from McLellan GJ, Rasmussen CA. Optical coherence tomography for the evaluation of retinal and optic nerve morphology in animal subjects: practical considerations. Veterinary Ophthalmology. Sep 2012;15 Suppl 2:13–28.).

Gonioscopy revealed open or slightly narrowed iridocorneal angles, with subtle dysplasia of the pectinate ligament that is normally characterized by sparse and delicate fibers that span the entrance to the ciliary cleft in cats (Fig 2). This feature is consistent with goniodysgenesis, supported by the extreme narrowing of the ciliary cleft reported previously on high resolution ultrasonography of the ocular anterior segment in affected animals [16]. Additional clinical features noted in affected animals include prominent, elongated ciliary processes, spherophakia, iris hypoplasia (Fig 3) and iridodonesis (iris trembling). These features are readily and consistently observed in cats over 4 months of age, but are not consistently appreciable in younger kittens. More pronounced clinical signs of lens instability, i.e. anterior or posterior lens subluxation and luxation (ectopia lentis) occur within the first two years of life in ~8% of affected eyes. Haab’s striae (a feature of corneal stretching) or corneal edema are observed in fewer than 10% of affected eyes. When present, corneal edema is generally associated with lens instability and corneal endothelial contact by an anterior luxated or subluxated lens. Mild to pronounced optic nerve head cupping can be appreciated on ophthalmoscopy in all affected cats from 6 months of age (Fig 4) and is consistent with histopathological findings (see below).

**Fig 5:**
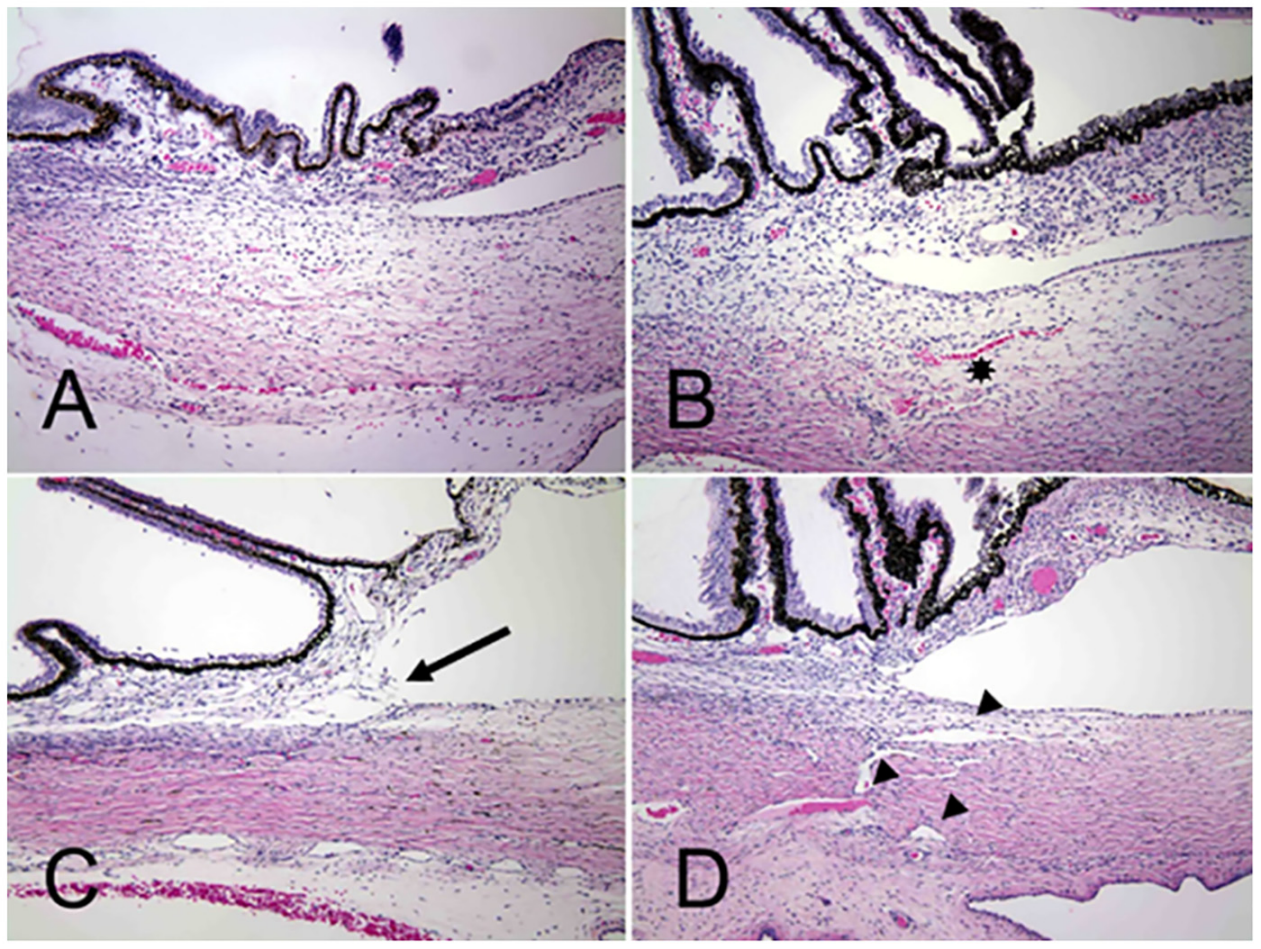
Arrested development of Aqueous Humor Outflow Pathways in Feline Primary Congenital Glaucoma. Photomicrographs of the iridocorneal angle in one day old (A and B) and 18 day old (C and D) cats. Affected cats (A and C) from the pedigree appear to develop fewer scleral vessels (asterisk) than normal and F1 out-crossed animals (B and D). Affected animals also fail to develop the normal vessels of the angular aqueous plexus (analogous to the canal of Schlemm in humans), collector channels and intrascleral venous plexus (arrowheads).

To characterize the morphologic abnormalities in the aqueous outflow pathway, sagittal histologic sections of enucleated eyes were evaluated by light microscopy. Eyes were obtained from 16 affected and 20 age-matched F1 out-crossed and unrelated control cats humanely euthanized from one day to 3 weeks of age. In the anterior segment a paucity of intra-scleral blood vessels, and hypoplasia of iris stroma and ciliary body, is evident at birth in affected cats (Fig 5). Elongation of the ciliary processes is a consistent finding in affected cats by 3 weeks of age. Post-natal development of the structures of the ciliary cleft, which should be well developed by three weeks of age in normal cats, [17] appears to be arrested in affected kittens. The uveal and corneo-scleral trabecular meshwork, angular aqueous plexus (analogous to Schlemm’s canal in human eyes), and associated vascular channels are rudimentary in affected animals and when visible, appear collapsed.

**Fig 6:**
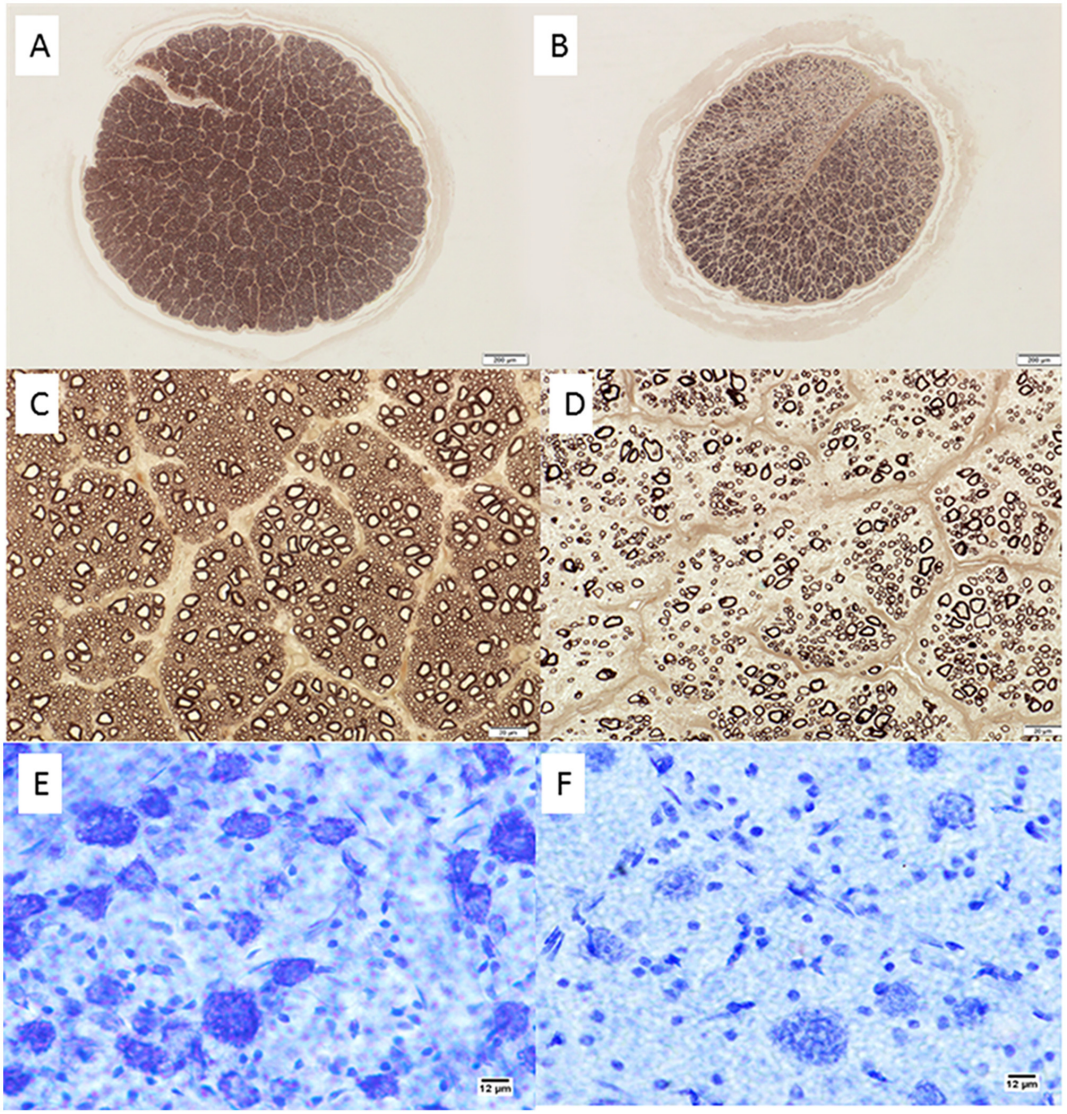
Retinal Ganglion Cell Loss in Cats With Primary Congenital Glaucoma. Photomicrographs showing representative optic nerve sections [A-D], stained with p-phenylenediamine to highlight axonal myelin sheaths, from 2 young adult cats. [A,C] normal cat (estimated 83,398 optic nerve axons); [B,D] typical PCG-affected cat with moderate degree of axon loss (estimated 30,365 optic nerve axons). Mid-peripheral retina in cresyl violet stained retinal whole mounts from these same two cats show relative loss of Nissl-stained retinal ganglion cell (RGC) somas of >12μm diameter in the ganglion cell layer of the retina in PCG (total RGC soma count in whole retina of 43,891)[E] relative to a normal cat [F] (estimated RGC soma count in whole retina of 123,833). Bar markers = 200μm (top row); 20μm (middle row), and 12μm (bottom row).

**Fig 7:**
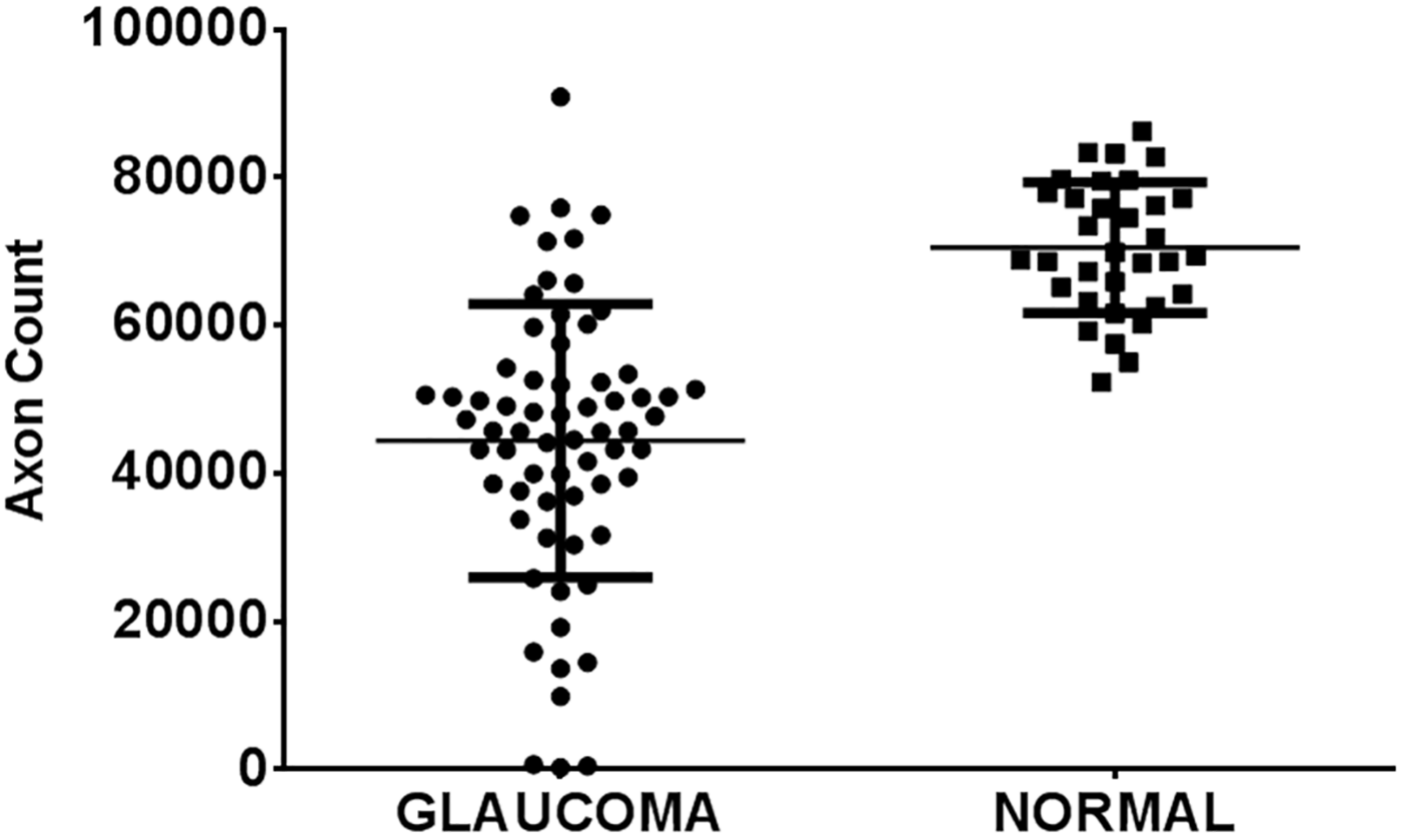
Optic Neuropathy in Feline Primary Congenital Glaucoma. Mean axon count in PCG cats (66 eyes of 38 cats aged 4 months to 8 years) is significantly lower (44,438 ± 2,272 [SEM]) than in normal cats (mean = 70,510 ± 1,535 in 33 eyes of 20 normal cats; p<0.0001, unpaired t-test). Lines represent mean and error bars represent SD.

Histological examination of optic nerve specimens derived from 66 eyes of 38 glaucomatous cats and 33 eyes of 20 normal cats that were 6 months of age or older confirmed that optic nerve head cupping observed clinically is accompanied by significant reduction in the number of optic nerve axons (Figs 6 and 7), consistent with a prior report of structural and functional abnormalities of the inner retina and optic nerve that were identified *in vivo* by optical coherence tomography and electrophysiology, respectively [18].

**Fig 8:**
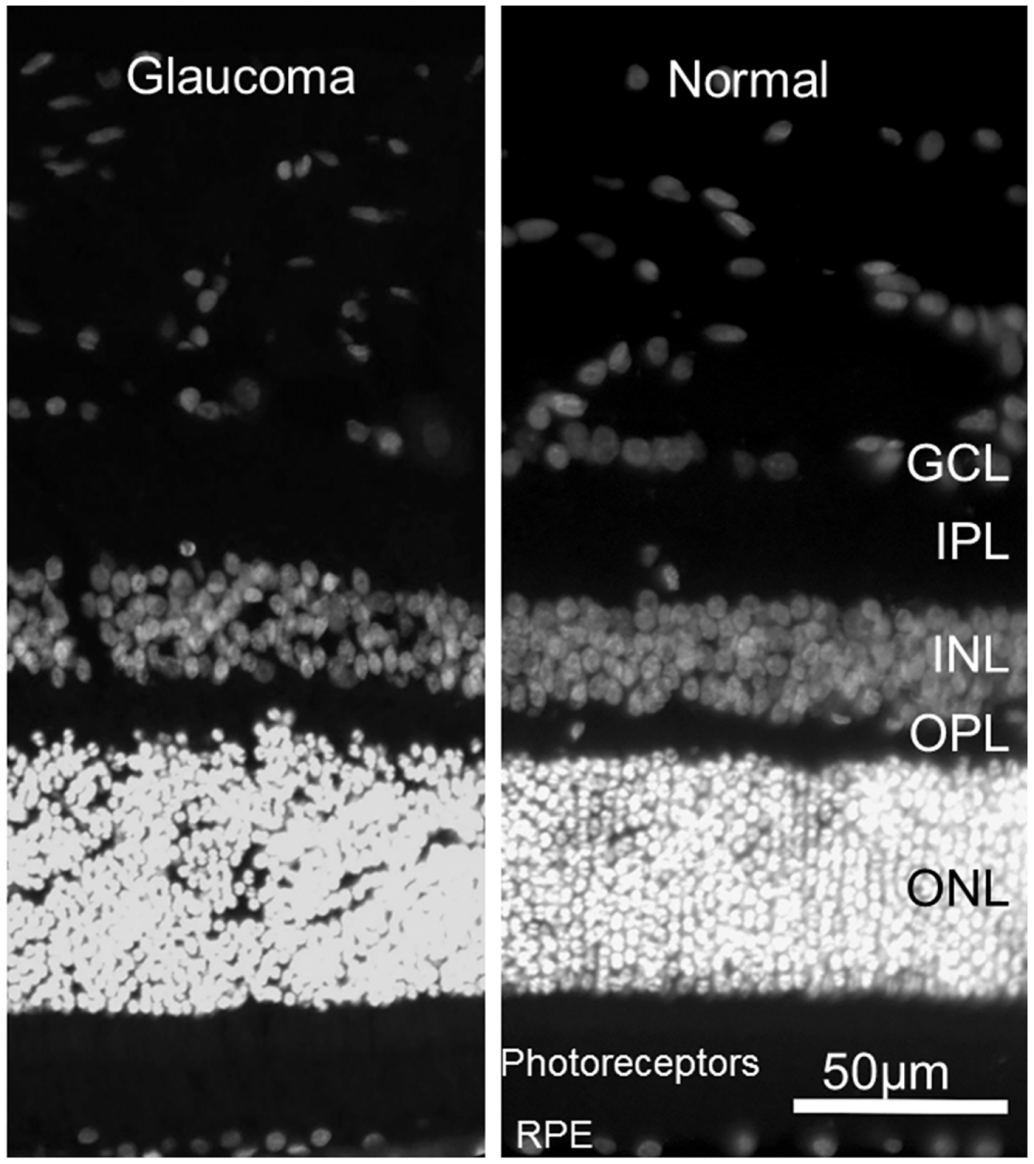
Preservation of Normal Outer Retinal Structure in Feline Primary Congenital Glaucoma. Representative fluorescence photomicrographs of DAPI stained 18 month old glaucomatous [A] and normal 6 month old [B] feline retinas. No significant difference in morphology of outer retinal layers is identified. (GCL = ganglion cell layer; IPL = inner plexiform layer; INL = inner nuclear layer; OPL = outer plexiform layer; ONL = outer nuclear layer; RPE = retinal pigment epithelium).

No significant outer retinal pathology is observed in the majority of cats with PCG (Fig 8). Thickness of the outer retinal layers is not significantly different from normal in young adult PCG cats (p>0.05, student t-test), except in those animals with severe disease and near total loss of retinal ganglion cell axons and somas.

### Inheritance pattern of feline PCG

**Fig 9:**
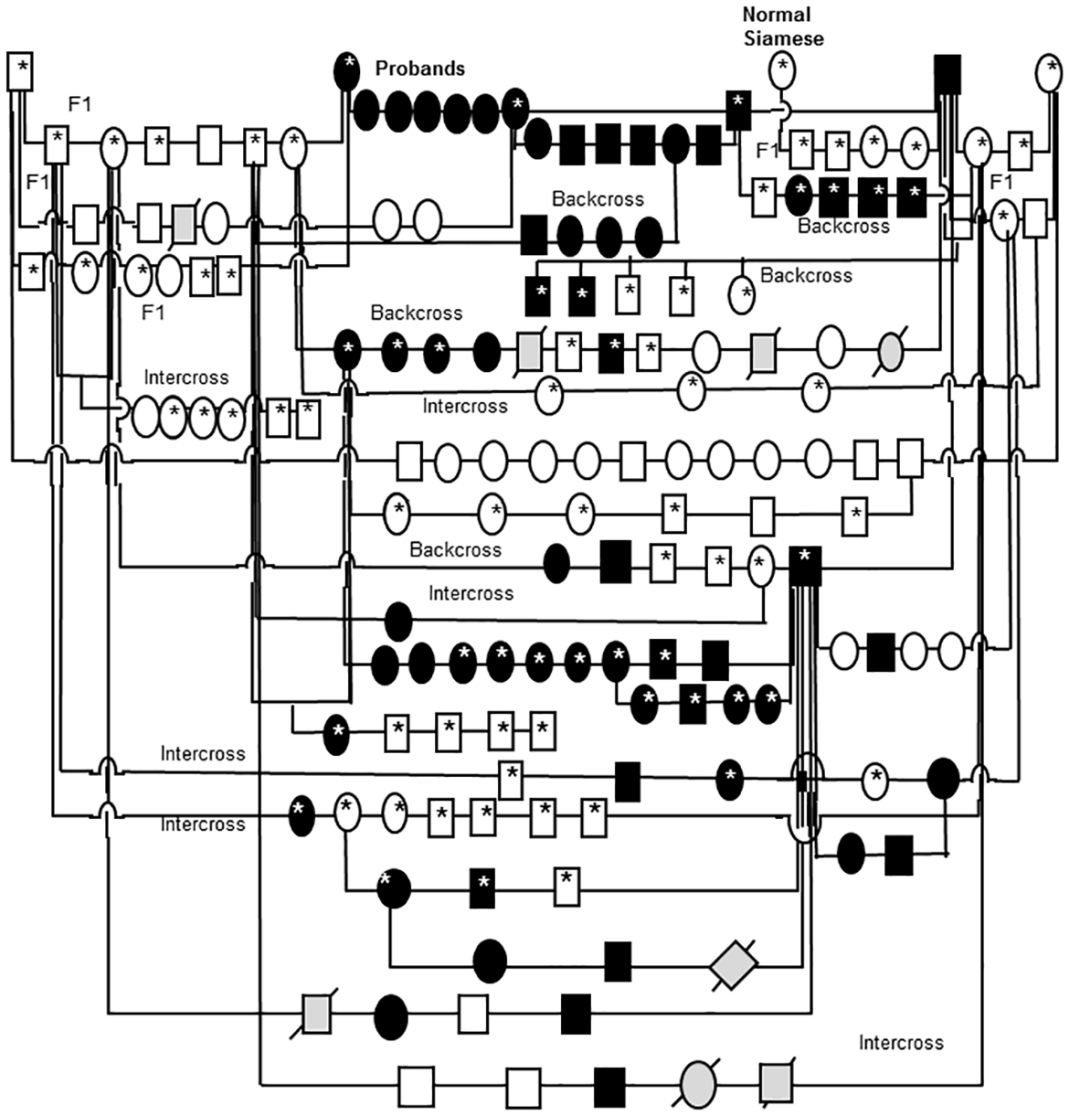
Feline congenital glaucoma pedigree. Additional pairings between affected and affected animals consistently produced all affected offspring but are not depicted in this informative pedigree. Females are depicted by circles and males by squares, diamonds indicate gender not recorded. Black shapes indicate that PCG phenotype confirmed by clinical examination conducted by a board certified veterinary ophthalmologist. Unfilled shapes indicate normal phenotype confirmed on clinical examination. Light gray shading with strike-through indicates that phenotype was not confirmed. Asterisks indicate those animals from which DNA samples were obtained and used in linkage analysis and/ or gene sequencing.

In order to determine the inheritance pattern of PCG in this pedigree of cats we established a breeding colony and created an expanded, informative pedigree (Fig 9). Beginning with the two affected Siamese founder cats and two unrelated, outbred domestic shorthaired cats we have produced over 400 offspring and over 100 litters. Dominant inheritance was excluded by pairing affected to affected (testing assumptions of heterozygosity for a dominant trait in either or both parents; p<0.025) and by pairing affected to normal, unrelated cats (p<0.0001). In 14 litters of offspring from carrier (F1) to carrier “intercross” or carrier to affected “backcross” pairings, the observed frequency of affected kittens is entirely consistent with that expected for autosomal recessive inheritance (p>0.5). Furthermore, all affected to affected pairings yielded only affected cats. Hence we conclude that the trait is fully penetrant and autosomal recessive.

Studies in mice have indicated that the ocular phenotype induced by disruption of the *CYP1B1* gene is considerably more pronounced in albino mice [19]. In order to evaluate whether the inheritance and phenotype of feline PCG could be dependent upon the Siamese genetic background (i.e. homozygosity for the *TYR-G301R* mutation responsible for the Siamese coat color phenotype, associated with ocular pigment dilution and blue irides) [20] [21], normal, domestic short-haired cats were bred to affected Siamese cats. Inter-crosses between clinically normal F1 out-crossed cats resulted in offspring with normal pigmentation and the glaucomatous phenotype. In these individuals the glaucomatous phenotype does not appear to differ in any way from that observed in Siamese cats, suggesting that this trait is independent of mutation in the *TYR* gene. Furthermore, the pairing of an affected Siamese founder with an unrelated, clinically normal Siamese cat yielded clinically normal Siamese offspring.

### Linkage Analysis

Using a subset of the animals we subsequently evaluated whether short tandem repeat (STR) markers tightly linked to candidate genes previously associated with glaucoma or anterior segment dysgenesis in humans exhibited linkage disequilibrium in the pedigree including *MYOC*, *FOXC1*, *CYP1B1*, *PITX2*, *OPTN*, *SH3PXD2B*, *PAX6*, and *LTBP2*. Only STR markers tightly linked with the *LTBP2* locus on feline chromosome B3 yielded results consistent with linkage. Consequently we examined this locus in greater detail using a total of 16 STR markers located within and flanking the *LTBP2* locus in the extended pedigree (Table 1). Significant linkages were established with an STR in the predicted 11^th^ intron of *LTBP2* (LOD 18.38, θ = 0.00), and with three additional STRs within *LTBP2* (LOD 8.3–17.2, –5.6, θ = 0.00) (Table 1). *S*everal STRs in flanking regions both 3’ and 5’ of the gene (Table 1) exhibited significant linkage with PCG, generally displaying decreasing LOD scores with increased distance from the gene (Table 1). These findings strongly implicate a PCG disease-causing locus located within the *LTBP2* gene on feline chromosome B3.

**Table 1:** Linkage Mapping of the Domestic Cat *LTBP2* Locus.

Primer Name	LOD	Theta	Position (Mb)*Cat chrB3
FCA391	1.7041	0.30	65,738,369–65,738,614
PS104	2.6909	0.30	103,122,602–103,122,815
PS110-2	5.4301	0.20	108,579,828–108,580,028
B3-110-1	5.3902	0.20	109,240,802–109,241,001
B3-118-1	6.1280	0.20	116,661,199–116,661,389
LTBP2_L2	8.2975	0.00	120,902,706–120,902,935
LTBP2-L1	14.6794	0.01	120,934,431–120,934,679
LTBP2-L5	18.3823	0.00	120,960,163–120,960,388
LTBP2-L4	17.1825	0.00	120,985,884–120,985,684
PS126	5.0796	0.20	122,720,591–122,720,776
PS130	6.2740	0.20	126,293,720–126,293,960
FCA88	5.8921	0.10	128,694,162–128,694,412
PS132	5.3587	0.10	128,203,904–128,204,079
PS134	5.4575	0.20	130,222,596–130,222,844
PS136	1.6201	0.20	132,098,251–132,098,494

* Markers are shown in genomic order along feline chromosome B3 based on the cat genome assembly version felCat5

**Fig 10:**
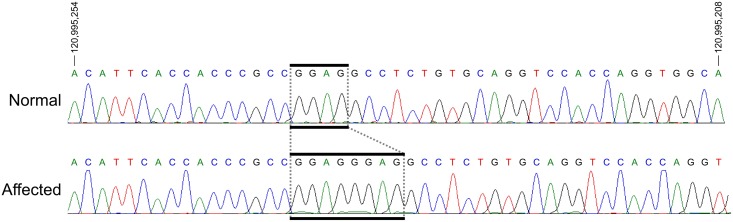
Sequences of Exon 8 of *LTBP2* in Normal Cat and in Primary Congenital Glaucoma. Sanger sequencing results of exon 8 of *LTBP2* in a normal (top) and a PCG-affected (bottom) individual. Analysis reveals a 4bp insertion in all affected individuals (boxed). Coordinates are for chromosome B3 from the felCat5 genome build.

Oligonucleotide primers flanking all exons of the feline *LTBP2* gene were then used to PCR amplify the entire coding region of this gene in several clinically affected and unaffected animals. Sequencing of these fragments revealed a 4-bp insertion in exon 8 located at chrB3: 120995236 (Fig 10). All examined affected animals (N = 14) were found to be homozygous whereas unaffected cats (N = 8) are either homozygous or heterozygous for the wild-type *LTBP2* sequence. Additionally, we detected three single nucleotide polymorphisms (SNPs) located at chrB3:120953711(G->C), chrB3:120974293 (C->T), and chrB3:120999159 (T->C) (FelCat5). In affected animals these SNPs appear to be in complete linkage disequilibrium with the insertion, however the last SNP is likely an unrelated polymorphism since it is also observed in some unaffected and unrelated cats. Furthermore, none of the SNPs results in a non-synonymous mutation and consequently it is unlikely that these changes result in the observed phenotype in affected cats.

The *LTBP2* mutation was not identified in archived DNA samples from 148 unaffected cats. This archive included 68 unrelated Siamese cats and 80 other cats representing 31 cat breeds, as previously reported [22, 23].

### *LTBP2* is transcribed in the eye of both normal and PCG-affected cats

In order to confirm the predicted mRNA sequence of feline LTBP2 and to determine the transcriptional consequences of this mutation we carried out whole transcriptome shotgun sequencing (RNAseq) of mRNA isolated from the iridocorneal angle of a young, PCG- affected cat and an age matched, unrelated healthy control cat. These data were analyzed to determine if multiple isoforms of the Latent Transforming Growth Factor-β Binding Protein 2 (LTBP2) exist and if transcripts for *LTBP2* can be detected in the anterior segment of affected animals.

The normal cat *LTBP2* sequence (S1 Fig) contains 11,523 nucleotides and encodes a protein of 1,823 amino acids with a predicted molecular weight of 195 kDa (GenBank accession number KT625609). The first 35 amino acids are likely removed during processing of the protein resulting in a molecular weight of 192.1 kDa [24]. The normal protein contains 13 Epidermal growth factor (EGF)-like domains as well as 3 Transforming Growth Factor (TGF)-binding domains, as predicted by the NCBI Conserved domain algorithm (http://www.ncbi.nlm.nih.gov/Structure/cdd/wrpsb.cgi) [25].

Phylogenetically the feline *LTBP2* protein is most closely related to that of other members of the order Carnivora such as the dog and ferret (94% and 92% identity over 100% length, respectively). The sequence homology to the human orthologue is 87% over 100% length and sequence conservation is slightly lower in mice (77% over 98% length) (S2 Fig).

**Fig 11:**
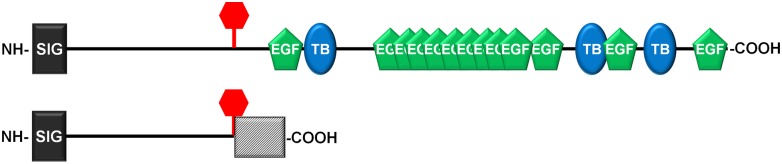
Predicted Effects of Mutation on Feline LTBP2. Schematic representation of the feline LTBP2 protein in normal (top) and affected cats (bottom). The sequence contains an amino terminal signal sequence (dark rectangle), 13 EGF-like calcium binding domains (green) and 3 TGF binding domains (blue). The insertion site causing a frameshift mutation in affected animals is marked by a vertical bar and the altered sequence caused by the frameshift is indicated by a hatched box.

The four bp insertion mutation detected in affected animals results in a frameshift mutation after nt 2002 of the cDNA sequence. Consequently the mutation induces a frameshift and a faulty amino acid sequence after residue 486 (S1 Fig). This aberrant sequence incorporates a further 114 incorrect amino acids before encountering a stop codon and being terminated. The mutated protein has a predicted molecular weight of 63.7 kDa and, if it is in fact translated, would lack all functional domains observed in the normal *LTBP2* gene product. (Fig 11)

**Fig 12:**
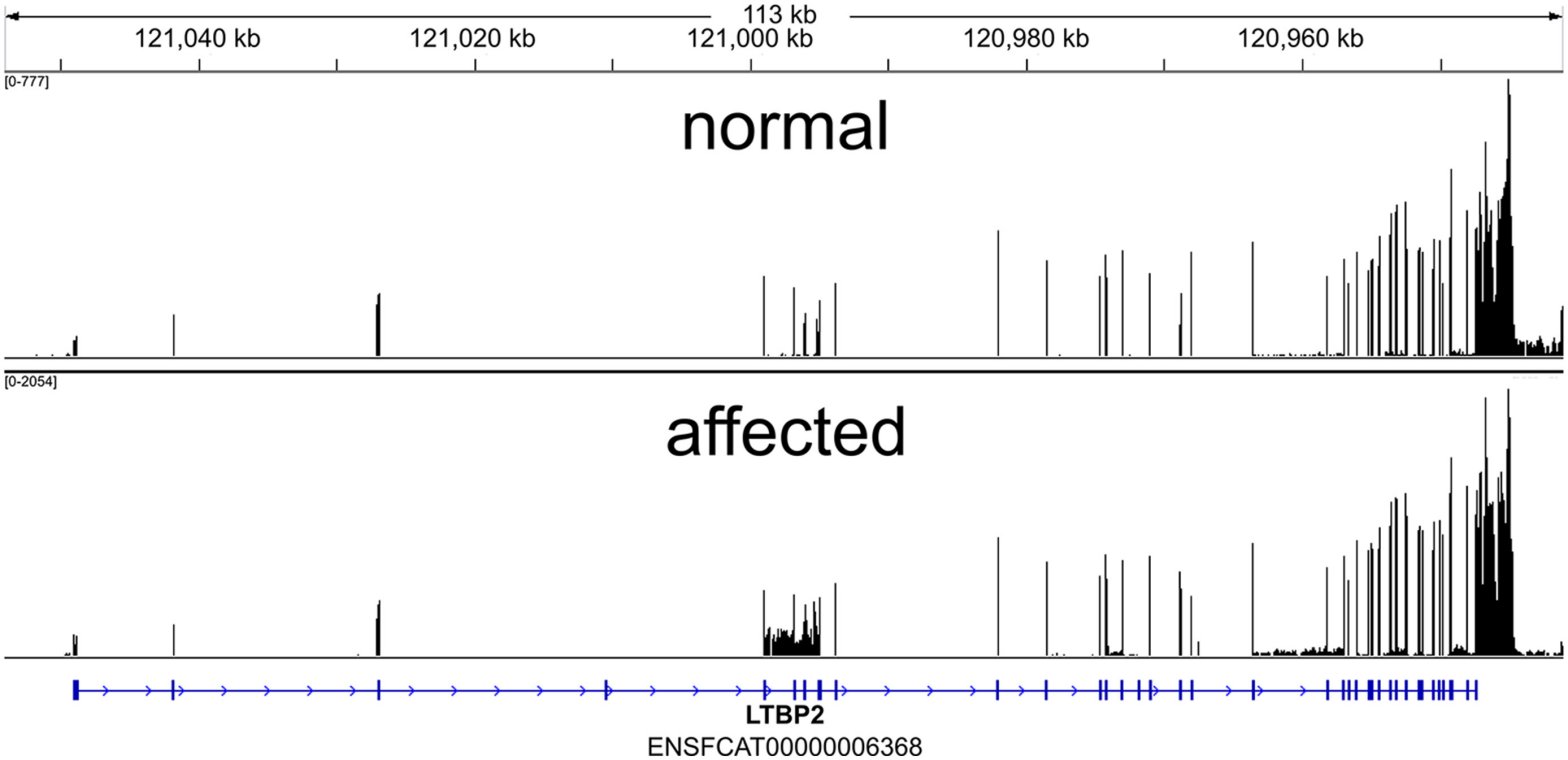
Coverage across Feline LTBP2 exons by RNA-Seq. RNA-Seq read coverage across the *LTBP2* exons within the cat genome. Coordinates are shown in genomic orientation. The numeric range in the top left indicates the minimum and maximum number of reads (FPKM-Fragments Per Kilobase of exon per Million) per base for the sample.

Transcriptional analysis suggested that there is only one major LTBP2 transcript in the anterior segment of cat eyes (GEO accession number GSE73263; http://www.ncbi.nlm.nih.gov/geo/query/acc.cgi?acc=GSE73263). While we cannot conclusively rule out the existence of splicing isoforms, our data indicate that these would be quite rare. LTBP2 mRNA was abundant in the tissue samples obtained from an affected cat and no evidence of skipped exons, incompletely removed intronic sequences, or preferential mRNA degradation was evident when compared to a healthy control (Fig 12).

## Discussion

In this manuscript we present linkage data and molecular genetics that identify a mutation associated with congenital glaucoma in a novel, spontaneous feline model of PCG. Affected cats exhibit elevated IOP early in life, globe enlargement, eventual optic nerve head cupping and axon loss, and other clinical and pathological features consistent with glaucoma. Breeding studies demonstrated an autosomal recessive mode of inheritance for feline PCG, as has been reported for familial congenital glaucoma in humans, which has greatest prevalence in populations with high rates of consanguinity [26]. The relatedness of our affected Siamese founder animals was not determined, although they were reportedly obtained from the same breeder.

Congenital glaucoma is rare in cats, although sporadic cases of congenital and goniodysgenetic glaucomas in Siamese, Persian, and domestic short-haired cats have been documented [10, 27–29]. There are no previous published reports of congenital glaucoma associated with spherophakia in Siamese cats. However, case descriptions have been published in the veterinary literature involving young Siamese cats with micro-spherophakia, in which IOP and optic nerve head appearance are not reported and these case reports from the USA and Europe appear to share many clinical features with the PCG-affected cats in our cohort [30, 31]. Thus, it is conceivable that the mutation responsible for this recessively inherited disease may have a wide geographic distribution within the Siamese breed and could be responsible for apparent predilection for glaucoma in this breed [32, 33]. Although the mutation was not detected in a preliminary screen of 148 archived DNA samples at the NCI’s Laboratory of Genomic Diversity that represented 32 breeds, including 68 Siamese cat samples, additional screening of the Siamese cat population for the disease causing mutation warrants consideration, in order to establish prevalence of this genetic condition within the breed and guide breeding practices to avoid the glaucoma phenotype.

One of the defining features of Siamese cats is their characteristic coat color which is caused by a *TYR-G301R* tyrosinase mutation in their genetic background [21]. Tyrosinase deficiency has been reported to increase the severity of developmental abnormalities of the aqueous outflow pathways in albino *cyp1b1* knockout mice [19]. We therefore considered it possible that severity of ocular phenotype was modified by ocular pigment dilution in our founding Siamese cohort. However, by out-crossing to domestic short-haired cats with conventional coat colors and subsequent back-crosses, we were able to reproduce an identical ocular phenotype in normally pigmented cats [14]. Consequently it appears that the disease causing mutation is highly penetrant and is not modified by tyrosinase deficiency or other factors in the Siamese cat genetic background.

Our breeding studies generated an informative pedigree that, coupled with advances in the feline genome project, identified significant linkage to the gene *LTBP2* on feline chromosome B3, and subsequent sequence analysis identified a 4-bp insertion in exon 8, introducing a frameshift and truncation of the protein which is likely causal for PCG in this pedigree. This finding is of particular importance since mutations in *LTBP2* have also been implicated in human congenital glaucoma [7, 15]. Pathogenic missense mutations have been reported, but human PCG patients are typically homozygous for insertion or deletion mutations leading to frameshift and premature truncation. These aberrations have been observed throughout the length of the *LTBP2* gene [7, 15, 34, 35]. Our RNA sequence analysis suggests that mutated *LTBP2* is transcribed and that the mRNA is not removed by nonsense-mediated decay. While unusual, escape from this control mechanism has been reported in other systems and may be related to the length of the open reading frame [36, 37]. Consequently, it is likely that mutated LTBP2 is synthesized in affected cats and possibly also in some human patients. This may lead to the accumulation of a truncated protein with an aberrant carboxy terminal domain, but it is also possible that these proteins are not retained within the cell and degraded [38]. These cats not only further establish a role for *LTBP2* as a glaucoma causing gene but also provide a spontaneous animal model allowing further investigation of the pathophysiological mechanisms of PCG as well as potential treatment approaches.

LTBP2 shares about 45% amino acid identity to LTBP1 but, unlike other LTBPs, does not appear to directly bind to latent forms of transforming growth factor β (TGFβ). Rather, it appears to provide structural support during microfibril assembly in the extracellular matrix (ECM) [39]. However, altered *LTBP2* expression, through its impact on assembly of micofibrils and ECM composition can also influence the bioavailability of TGFβ [40, 41], which is known to play a complex and important role in glaucoma pathogenesis [42–47]. The spectrum of clinical disease associated with mutations in *LTBP2* is rather broad and includes megalocornea, microspherophakia and ectopia lentis [34, 48, 49], as well as congenital glaucoma [7, 15, 35]. A role has also been proposed for *LTBP2* mutations in adult onset open angle glaucomas [50]. Generalized ECM abnormalities resembling Marfan disease and Weill-Marchesani syndrome have been observed [34, 35, 51] Further, *LTBP2* has been identified as a potential candidate biomarker for fracture risk [52], death associated with pulmonary disease with acute dyspnea [53] and adverse renal events in humans [54]. No clinical signs consistent with Weill-Marchesani syndrome, such as short stature, or of Marfan syndrome, such as tall stature, pes excavatum or aortic aneurysm, have been identified on either clinical or pathological examination in any cats within this colony. Thus, the disease phenotype appears to be largely confined to ocular disease in affected cats. However, longer-term, detailed clinical and histopathologic evaluation of systemic phenotype is ongoing.

It is noteworthy that findings in this cat model differ from those described in knock-out mice. Early embryonic lethality was previously ascribed to *ltbp2* deletion in mice [55], but this finding was not duplicated in a more recent study [38]. In our colony, *LTBP2* mutant cats are as fertile as control animals and the resulting offspring appear healthy apart from the ocular phenotype. Thus our studies do not indicate that lack of *LTBP2* is lethal. Furthermore, studies of Inoue, et al. [38], using *ltbp2* null mice, provided strong experimental support for a role of this protein in ciliary zonule microfibril assembly, a role that derives further support from our observation of lens zonular instability in affected cats in our pedigree. However, the conditional knockout mice demonstrated consistent lens luxation, which is relatively infrequent among affected animals in our feline colony. Finally glaucoma was not identified and pronounced abnormalities in the aqueous outflow pathways were not apparent in the mouse model [38]. The reason for these differences among models is not known but could be related to the nature of the mutation. Specifically, it is conceivable that affected cats synthesize a truncated LTBP2 that retains some functionality that is lost in knock-out mice. Additionally, species differences related to structure of the eye and longevity may be a reason for the observed disparities.

The extremely high prevalence of glaucoma and the developmental abnormalities we identified in the aqueous outflow pathways of affected cats strongly suggest that caution should be exercised in attributing glaucoma pathogenesis in patients with *LTBP2* mutations solely to lens instability, particularly as phenotypic heterogeneity has been described in human patients, even between individuals with the same mutation in *LTBP2* [35]. Thus, genotype may not be fully predictive of phenotype in families harboring *LTBP2* mutations and both primary and secondary glaucoma may occur. As the surgical management of primary congenital glaucoma and lens-associated glaucomas are very different, phenotypic heterogeneity observed in human families and among affected cats in our large pedigree underscores a need for individualized treatment planning in patients with pathogenic *LTBP2* mutations.

## Materials and Methods

### Animals

All animal studies were carried out in adherence with the ARVO Statement for the Use of Animals in Ophthalmic and Vision Research and in strict accordance with the recommendations in the Guide for the Care and Use of Laboratory Animals of the National Research Council and the National Institutes of Health. All studies were approved by the Iowa State University and University of Wisconsin-Madison Institutional Animal Care and Use Committees. Cats were housed and managed in a laboratory animal facility under controlled environmental conditions with 12 hour light cycles, in social groups of 2–15 cats provided with appropriate enrichment. Cats were fed high quality, commercially available feline dry diets appropriate to their life stage and health status. The colony was founded as described [14], by two glaucomatous Siamese cats and their glaucomatous offspring. Outcrosses were obtained, pairing glaucomatous cats with two normal domestic shorthaired cats and a normal Siamese cat. Disease status was determined as described below, by clinical evaluation in conscious, lightly restrained cats. Mortality rates in this study were much lower than in the feline population at large, and as a result necropsy was not routinely conducted. In addition to neonatal mortalities, rare animals met designated endpoints established for pain, discomfort and illness, as a result of illnesses and injuries unrelated to the glaucoma phenotype. Animals meeting these criteria were euthanized at the clinical discretion of the Institutions’ veterinary staff. Humane endpoints included failure to thrive, progressive weight loss not alleviated by supplemental feeding, or occasional non—ocular congenital defects.

### Clinical evaluation

All 147 animals surviving to 6 weeks or older were evaluated by a board certified veterinary ophthalmologist by slit-lamp biomicroscopy (SL14, Kowa, Japan); IOP measurement by applanation tonometry (Tono-pen XL, Medtronic, Jacksonville, FL) following the application of 0.5% proparacaine (Akorn Inc., Lake Forest, IL), or rebound tonometry (TonoVet, ICare oy, Finland) [56]; direct ophthalmoscopy (Welch Allyn Inc., Skaneateles Falls, NY), and indirect ophthalmoscopy (Keeler, Broomall, PA) following topical administration of 0.5% tropicamide (Bausch and Lomb, Inc., Tampa, FL, USA).

### Histopathology

#### Anterior segment development

Eyes were obtained immediately following humane euthanasia by intravenous administration of barbiturate overdose, from 16 homozygous, affected cats; 12 out-crossed F1 cats, and 8 age-matched domestic short-haired control cats ranging in age from 1 to 70 days. Eyes were fixed immediately in 4% paraformaldehyde, embedded in paraffin, sectioned to a thickness of 5 μm, and stained with Periodic acid-Schiff (PAS) or hematoxylin / eosin then examined by light microscopy.

#### Evaluation of optic nerves and retinas

To validate our clinical findings, optic nerve tissues were also obtained from eyes of 58 cats ranging in age from 4 months to 8 years that were perfusion or drop-fixed with 4% paraformaldehyde, immediately following euthanasia as described above, over the course of a 10 year period, for various reasons not directly related to the current study. Optic nerves were post-fixed with glutaraldehyde, resin embedded, osmicated, semi-thin sectioned and stained with Richardson’s stain or p-phenylenediamine (PPD). Axons were quantified in PPD stained sections using a semi-automated targeted method as previously described [57]. Whole retinas from 2 normal and 6 PCG cats were mounted on gelatin coated slides and Nissl-stained with cresyl violet for identification of RGCs (distinguished from glia and displaced amacrine cells based on morphologic criteria) and manual counting by light microscopy [58, 59]. Thicknesses of the outer retinal layers in DAPI stained sagittal cryosections of peri-papillary retina from 3 normal and 5 young adult PCG cats were measured in fluorescence photomicrographs using commercially available image analysis software (AxioVision 4.8, Carl Zeiss Microscopy).

#### Breeding studies

The colony was founded by two glaucomatous Siamese cats and their glaucomatous offspring. Outcrosses were obtained by pairings with normal domestic shorthaired laboratory cats. Disease status was determined by clinical evaluation, as described below. Disease status was determined in 25 F1 out-crossed cats; in 30 F1 back-crossed cats, and in total over 60 PCG-affected cats were evaluated. Dominant inheritance was assessed by Chi-squared analysis for goodness of fit of 4 pairings, involving 3 affected Siamese cats and 2 clinically normal, unrelated domestic short-haired cats. In order to evaluate whether the inheritance of feline congenital glaucoma could be dominant on a Siamese genetic background, i.e. homozygosity for the *TYR-G301R* mutation responsible for the Siamese coat color phenotype [21], a normal, unrelated Siamese female was bred to an affected Siamese male. Recessive inheritance was examined by Chi-squared analysis of seven back-crosses, pairing clinically normal F1 out-crossed females with clinically affected Siamese males; and five pairings of F1 out-crossed males and females.

### Candidate Gene and Linkage Analysis

For molecular genetic studies, DNA was routinely isolated from feline blood or tissue samples according to manufacturer’s protocols using the QIAampDNA Mini Kit (Qiagen; http://www.quiagen.com). DNA was quantified using the NanoDrop spectrophotometric method (http://www.nanodrop.com).

Linkage analysis of candidate loci was carried out using short tandem repeat markers (STRs) to genotype an informative subset of the larger PCG pedigree (S1 Table). Candidate genes included 8 genes previously associated with congenital or other heritable forms of glaucoma, or ocular anomalies in humans (*MYOC* [56], *FOXC1* [60, 61], *CYP1B1* [6], *PITX2* [62], *OPTN* [63], *SH3PXD2B* [64], *LTBP2* [7], and *PAX6* [65]). DNA samples were quantified, inspected on a gel, and genotyped using a forensic panel, the MEOWPLEX [22], to verify pedigree structure and confirm Mendelian inheritance. Linkage analysis computations were performed with SUPERLINK (http://bioinfo.cs.technion.ac.il/superlink-online/) using a fully penetrant, autosomal disease model with a disease allele frequency of 0.01. Marker allele frequencies were set as equal. Primers were designed tightly linked to the 8 listed candidate genes using the cat genomic browser GARField [66]. Primers were tested to determine informativeness of the STRs and for yield of expected amplicon length (S1 Table). Amplification of the STRs was performed by a touchdown PCR reaction protocol as described [22]. PCR products were fluorescently labeled using M13 tailed primers as described [67]. Products were analyzed on an Applied Biosystems Model 3100 genetic analyzer with Genescan version 3.7 and Genotyper version 2.5 software (http://www.appliedbiosystems.com).

The feline *LTBP2* mRNA sequence was deduced by BLAST alignment of the respective human cDNA sequence to the feline genome (felCat4). Portions of the exons not represented in the database were isolated by one-sided PCR, cloned and sequenced. Sequence information was used to design PCR primer pairs that allowed the amplification of all exons of the gene (S1 Table). Following amplification, the genomic sequences from affected and unaffected animals were determined by Sanger sequence analysis and evaluated for the presence of sequence variations indicative of an involvement of the candidate gene in the development of the phenotype.

#### *LTBP2* transcriptional analysis

Total RNA was extracted from the iridocorneal angle and ciliary body, including the adjacent sclera and trabecular meshwork of an affected 2 month-old cat and an unrelated, age-matched healthy control. After confirmation of RNA integrity both samples were transcriptionally profiled using the HiSeq sequencing system (Illumina, San Diego, CA, http://www.illumina.com). The resulting sequence reads were aligned to *Felis catus* genome build felCat5 using Tophat software (ver. 2.0.9 http://ccb.jhu.edu/software/tophat/index.shtml) [68]. Run parameters were: Tophat (ver. 2.0.9) (—max-insertion-length 4,—read-mismatches 2,—read-gap-length 4,—read-edit-dist 4, -G with Ensembl gene predictions)

Transcript abundance and differential expression were calculated using the software Cufflinks (ver. 2.1.1; http://cufflinks.cbcb.umd.edu/)[68], and cummeRbund (ver. 2.4.1; http://compbio.mit.edu/cummeRbund/)[69]. Analysis parameters for Cufflinks were (—max-bundle-frags 20000000).

To generate cDNA sequences, exon coordinates for each isoform identified were exported from cummeRbund. Consensus variants within these regions were called using samtools mpileup (ver. 0.1.18) and bcftools (ver. 0.1.17-dev)[70]. This produces a single variant call at each position based on the most common allele observed in the RNA-Seq reads. Variants with QUAL value ≥ 100 and exon coordinates were passed on to GATK FastaAlternateReferenceMaker (ver. 2.5-2-gf57256b) to produce mRNA sequences. Short regions that remained unresolved in felCat5 were bridged by manual incremental alignments of unmapped sequence reads to the obtained consensus sequence.

cDNA sequence translations to amino acid sequences were carried out using ExPASy (http://www.expasy.org) tools and protein domains were mapped using the NCBI conserved domains server (http://www.ncbi.nlm.nih.gov/cdd/). Sequences with substantial open reading frames were aligned using the Clustal Omega implementation at Uniprot (http://www.ebi.ac.uk/Tools/msa/clustalo/)[71].

## Supporting Information

S1 Fig Complete cDNA and amino acid sequence of feline LTBP2. The initiation codon is shaded yellow. The 4-bp sequence, which is duplicated in affected animals, is included in the cDNA sequence and shaded red. Amino acid sequences of healthy (LTBP2) and affected (affec) animals are included.
(DOCX)

S2 Fig Sequence homology of feline *LTBP2* to the human orthologue is 87% over 100% length. Sequence homology to human *LTBP2* is slightly lower in mice (77% over 98% length).
(DOCX)

S1 Table Short tandem repeat markers (STRs) used for 7 candidate genes and for *LTBP2* in linkage analysis to genotype an informative subset of the larger PCG pedigree. Primers were tested to determine informativeness of the STRs and for yield of expected amplicon length. The third tab in this spreadsheet shows PCR primer pairs that enabled the amplification of all exons of the feline *LTBP2* gene.
(XLSX)

## Abbreviations

RGC: retinal ganglion cell
IOP: intraocular pressure
PCG: primary congenital glaucoma
TYR: tyrosinase
LTBP2: Latent Transforming Growth Factor-β Binding Protein 2
STR: short tandem repeats
PCR: polymerase chain reaction
