# Correction: A Mutation in LTBP2 Causes Congenital Glaucoma in Domestic Cats (*Felis catus*)

**DOI:** 10.1371/journal.pone.0161517

**Published:** 2016-08-18

**Authors:** Markus H. Kuehn, Koren A. Lipsett, Marilyn Menotti-Raymond, S. Scott Whitmore, Todd E. Scheetz, Victor A. David, Stephen J. O'Brien, Zhongyuan Zhao, Jackie K. Jens, Elizabeth M. Snella, N. Matthew Ellinwood, Gillian J. McLellan

In Fig 1, the numbers (n) of subjects are missing from the bars of the bar graph. Please see corrected Fig 1 here.

**Fig 1:**
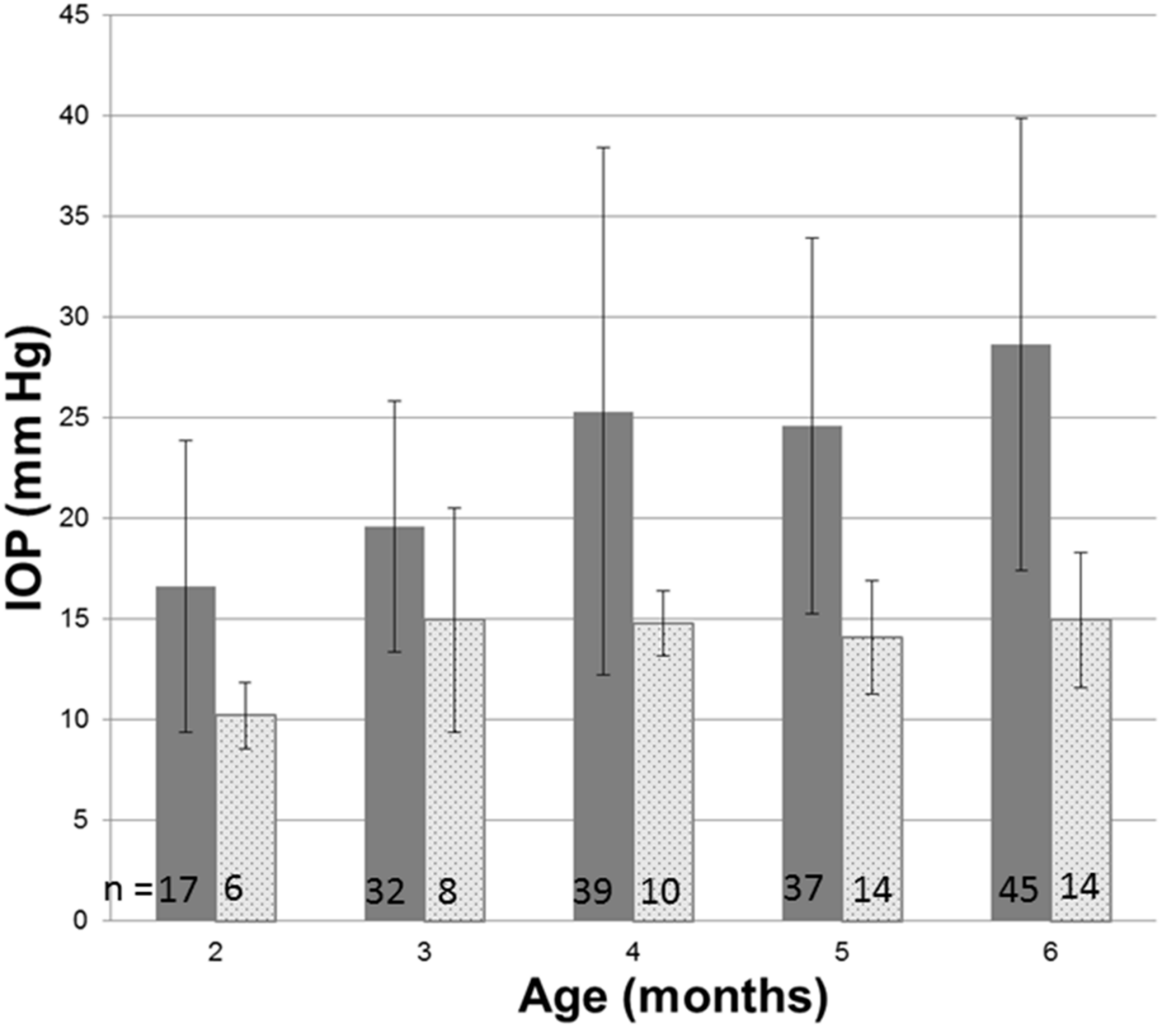
Intraocular pressure in glaucomatous and normal cats. Intraocular pressure measured by rebound tonometry (TonoVet, ICare oy, Finland) was significantly greater in PCG (solid bars) than in normal cats (patterned bars) at all ages from 2 months of age (ANOVA with Tukey-Kramer Multiple multiple comparisons post-test, p<0.05). Error bars represent standard deviation. Numbers (n) of subjects in each age group are indicated on each column.

## References

[1] Kuehn MH, Lipsett KA, Menotti-Raymond M, Whitmore SS, Scheetz TE, David VA, et al (2016) A Mutation in LTBP2 Causes Congenital Glaucoma in Domestic Cats (*Felis catus*). PLoS ONE 11(5): e0154412 doi: 10.1371/journal.pone.0154412 27149523 10.1371/journal.pone.0154412 PMC4858209

