# Supplementary material for: A Mutation in LTBP2 Causes Congenital Glaucoma in Domestic Cats (Felis catus)
Source: PLoS One. 2016 May 5;11(5):e0154412. doi: 10.1371/journal.pone.0154412 (PMC4858209; doi:10.1371/journal.pone.0154412)
Supplement: S1 Fig — The initiation codon is shaded yellow. The 4-bp sequence, which is duplicated in affected animals, is included in the cDNA sequence and shaded red. Amino acid sequences of healthy (LTBP2) and affected (affec) animals are included. (DOCX) [file pone.0154412.s001.docx]

0001: gttcgagcttgcccagcacgagagaaaggcgaccaggtgcccccgtcccggcaggcgtcgtctcccccgccagccgtcgggtcagctctg

0091: cgctgcccccagctcctcacgggttaacccccctcgccgcccgctcgcgtcccggtgccaaagtgggtgttgctggaattcctctctccc

0181: tctcccgtaatgagggggctgagctgtccctccgaggagggggcatgatgtagataaaagagacgaaaaaacagggggaagtttccaaaa

0271: ataaaagcgtccgtctccccttcagaaggccgcagagaccgggagaaggcgcgaaaagtgcagcggcggtgcgcacccggctggagacga

0361: gcggacgccaggctcggggcgcccagccccgcagcgctcacgccgcccagccctcgcccggggaaggctggctcggagcccctagaccag

0451: gagtagcgggccgccggcaggggagcggagcgcaggcgccgcaagcccccggggctgttccccgcgctcaccgctctccagtcgcccccg

0541: gagccatgaggcccccgaccacccaccgctgcaccgggcgcgccctgcggaacccctgcgggagcctcctggccctcaccctggctctct

LTBP2 M R P P T T H R C T G R A L R N P C G S L L A L T L A L F

affec M R P P T T H R C T G R A L R N P C G S L L A L T L A L F

0631: tcgtgggcatgggtcacgctcagagggacccggtagaaagatccgagccggctggcagggaggcgaaccggctgcggcgccccgggggca

LTBP2 V G M G H A Q R D P V E R S E P A G R E A N R L R R P G G S

affec V G M G H A Q R D P V E R S E P A G R E A N R L R R P G G S

0721: gtcaccccgccgcggctgccgccaaggtgtacagtctgttccgagagcaggacgcgcccgtcccgggcttgccgcccacggagcgggccc

LTBP2 H P A A A A A K V Y S L F R E Q D A P V P G L P P T E R A Q

affec H P A A A A A K V Y S L F R E Q D A P V P G L P P T E R A Q

0811: aactcggctgggggagcgcccggaggcccgccaacgcgctggccaggaggccgccccgcgcgcagcagccgcgccgagcccagccacctg

LTBP2 L G W G S A R R P A N A L A R R P P R A Q Q P R R A Q P P A

affec L G W G S A R R P A N A L A R R P P R A Q Q P R R A Q P P A

0901: cgcagacctggagaagcagtcccctgggccagcagcagcccgcagtccgcgcccgggccgccccggctctcccgcgcctcggaaccccgc

LTBP2 Q T W R S S P L G Q Q Q P A V R A R A A P A L P R L G T P Q

affec Q T W R S S P L G Q Q Q P A V R A R A A P A L P R L G T P Q

0991: agcggcccggggctgcgcccccaaccccgccgcgagggcgacttacggggaggaatgtctgcgggggacagtgctgcccgggatggacga

LTBP2 R P G A A P P T P P R G R L T G R N V C G G Q C C P G W T T

affec R P G A A P P T P P R G R L T G R N V C G G Q C C P G W T T

1081: cggcgaacagcaccaaccactgtatcaaacctgtgtgccagccgccctgccagaaccggggatcctgtagccggccccagctctgcgtgt

LTBP2 A N S T N H C I K P V C Q P P C Q N R G S C S R P Q L C V C

affec A N S T N H C I K P V C Q P P C Q N R G S C S R P Q L C V C

1171: gccgctccggcttccggggagcccgctgcgaggaggtcattcccgaggaggaatttgacccccagaactcgaggccagcgccccggcgct

LTBP2 R S G F R G A R C E E V I P E E E F D P Q N S R P A P R R S

affec A E G S P N P R G S S A A R E S T T T P R T R S L A S Q L P

1261: cagccgaggggtcacccaacccacgcgggagcagtgcagccagagaaagcaccacgacccccagaacacgctcactggcctcgcagctgc

LTBP2 A E G S P N P R G S S A A R E S T T T P R T R S L A S Q L P

affec A E G S P N P R G S S A A R E S T T T P R T R S L A S Q L P

1351: cgccggccaggagtctgcccccagccaggaccctgagtgggctcagccagacgcagccctcccagcagcatgtggggttgtcccggacgg

LTBP2 P A R S L P P A R T L S G L S Q T Q P S Q Q H V G L S R T A

affec P A R S L P P A R T L S G L S Q T Q P S Q Q H V G L S R T A

1441: cccggctttacccaaccaccgcagccagcggtcagctcacctccaatgccctgcccgcagggccaggccttgagcggagggatggcaccc

LTBP2 R L Y P T T A A S G Q L T S N A L P A G P G L E R R D G T Q

affec R L Y P T T A A S G Q L T S N A L P A G P G L E R R D G T Q

1531: agcaggcggcgtatctggaccacccgtcatccccctgggggctgaacctcactgagaaaatcaagaagatcaagatcgtcttcactccca

LTBP2 Q A A Y L D H P S S P W G L N L T E K I K K I K I V F T P T

affec Q A A Y L D H P S S P W G L N L T E K I K K I K I V F T P T

1621: ccatctgcaagcagacttgtgcccgtgggcactgctccaacagctgtgaacgcggcgacaccaccaccctgtatagccagggtggccacg

LTBP2 I C K Q T C A R G H C S N S C E R G D T T T L Y S Q G G H G

affec I C K Q T C A R G H C S N S C E R G D T T T L Y S Q G G H G

1711: ggcatgaccccaagtctggcttccgaatctatttctgccagatcccctgcctgaacggaggccgctgcatcggcagggatgagtgctggt

LTBP2 H D P K S G F R I Y F C Q I P C L N G G R C I G R D E C W C

affec H D P K S G F R I Y F C Q I P C L N G G R C I G R D E C W C

1801: gccccaccaactccactgggaagttctgccacctgccggccccgaatctggacaaggggcctcccgagaggggctcccgccacagggccc

LTBP2 P T N S T G K F C H L P A P N L D K G P P E R G S R H R A L

affec P T N S T G K F C H L P A P N L D K G P P E R G S R H R A L

1891: tgctggaagtccccttgaggcagtccaccttcacactgcctctctccaaccagctggcctccgtgaacccctccttggtgaaggtgcaca

LTBP2 L E V P L R Q S T F T L P L S N Q L A S V N P S L V K V H I

affec L E V P L R Q S T F T L P L S N Q L A S V N P S L V K V H I

1981: ttcaccacccgccggagggaggcctctgtgcaggtccaccaggtggcacgggtgcggggcgaggccccggaggagaacagcgtggagacc

LTBP2 H H P P E ****A S V Q V H Q V A R V R G E A P E E N S V E T

affec H H P P E G G L C A G P P G G T G A G R G P G G E Q R G D Q

2071: agaccctcgccccggctccccgccagcccccgccacagccactgggacagcaacagcatccccgcccggtctggagaggcccctcagccc

LTBP2 R P S P R L P A S P R H S H W D S N S I P A R S G E A P Q P

affec T L A P A P R Q P P P Q P L G Q Q Q H P R P V W R G P S A P

2161: ccgcccccagcagcacccaggcctccgggactgctgggccgctgttatctgagctctgtgaacggacagtgtgccaaccccttgctggag

LTBP2 P P P A A P R P P G L L G R C Y L S S V N G Q C A N P L L E

affec A P S S T Q A S G T A G P L L S E L C E R T V C Q P L A G A

2251: ctgactgcccaagaggattgctgcggcagcgtgggagccttctggggggtgacctcgtgtgccccgtgcccacccagaccagcctcgccg

LTBP2 L T A Q E D C C G S V G A F W G V T S C A P C P P R P A S P

affec D C P R G L L R Q R G S L L G G D L V C P V P T Q T S L A G

2341: gtggttgaaaatggccagctggagtgtccccaagggtacaagagactgaatctcactcactgccaagatatcaacgagtgcctgaccctg

LTBP2 V V E N G Q L E C P Q G Y K R L N L T H C Q D I N E C L T L

affec G STOP

2431: ggcctgtgcaaggactcggaatgcgtgaacaccaggggaagttacctatgcacctgcaggcctggtctcatgctggacccgtccaggagc

LTBP2 G L C K D S E C V N T R G S Y L C T C R P G L M L D P S R S

2521: cgctgtgtgtccgacaaggccgtctccatgcagcaggggctgtgctaccggtcgctgggggctggcacctgcaccctgcctttggcccag

LTBP2 R C V S D K A V S M Q Q G L C Y R S L G A G T C T L P L A Q

2611: agcatcaccaagcagatctgctgctgcagccgagtgggcaaagcttggggcagcaagtgtgagagatgccccctccctggcacagagggc

LTBP2 S I T K Q I C C C S R V G K A W G S K C E R C P L P G T E G

2701: ttcagggagatctgccctgctggccacggctacacctactcgagctcacatatccgcctggccatgaggaaagccgaggaggaggaactg

LTBP2 F R E I C P A G H G Y T Y S S S H I R L A M R K A E E E E L

2791: gccaggccctccagggagcaagcgcagaagagctatgggaccctgctcgggccagcggagaggcagccactccgggcagtcactgacacc

LTBP2 A R P S R E Q A Q K S Y G T L L G P A E R Q P L R A V T D T

2881: tggctggaggccgagaccatccctgacaagggagactcccaggctggccaggtcacaaccagtgttacccaagtacctgcctgggtccca

LTBP2 W L E A E T I P D K G D S Q A G Q V T T S V T Q V P A W V P

2971: ggcaatgccacagaaagaccaacaccaccactgcctggacaggagattccagacaacccggaagaagagcgagtgaccaccccccatgat

LTBP2 G N A T E R P T P P L P G Q E I P D N P E E E R V T T P H D

3061: gggctggaggcccggggcccctcaggcattgaccggtgcgccactggagccaccaacatctgcggccctggaacctgcgtgaccctcccg

LTBP2 G L E A R G P S G I D R C A T G A T N I C G P G T C V T L P

3151: gacgggtacaaatgtctctgcagccccggctaccagctgcaccccagccaggcctactgcacggatgacaacgagtgtctgagggacccc

LTBP2 D G Y K C L C S P G Y Q L H P S Q A Y C T D D N E C L R D P

3241: tgcgctggaagagggcggtgtgtcaaccgagtgggttcctattcctgcttctgctaccctggctacacgctggccacctcggggacgacg

LTBP2 C A G R G R C V N R V G S Y S C F C Y P G Y T L A T S G T T

3331: caggaatgtcaagacatagacgagtgtgagcagccaggggtgtgcagcgggggacaatgcaccaatacagagggttcgtacgactgccag

LTBP2 Q E C Q D I D E C E Q P G V C S G G Q C T N T E G S Y D C Q

3421: tgtgaccagggctacatcatggtcaggaagggacactgtcaagatatcaacgaatgccgtcaccctggcacctgcccggatgggagatgc

LTBP2 C D Q G Y I M V R K G H C Q D I N E C R H P G T C P D G R C

3511: gtcaactcccccggctcctacacttgcctagcctgcgaggagggctacaggggccagagcgggagctgtgtagatgtaaatgagtgtctg

LTBP2 V N S P G S Y T C L A C E E G Y R G Q S G S C V D V N E C L

3601: acccctggggtctgcgcccatggaaagtgcatcaacctggagggctcctttagatgctcttgtgagccgggctatgaggtcacctcagat

LTBP2 T P G V C A H G K C I N L E G S F R C S C E P G Y E V T S D

3691: gagaagggctgccaagatgtcgacgagtgtgccagccgggcctcgtgccccacgggcctctgcctcaacactgagggctccttcacctgc

LTBP2 E K G C Q D V D E C A S R A S C P T G L C L N T E G S F T C

3781: tccgcctgcgagagtgggtactgggtgaatgaagacggcactgcctgtgaagacctagacgagtgcgcctttccgggagtctgcccctcg

LTBP2 S A C E S G Y W V N E D G T A C E D L D E C A F P G V C P S

3871: ggagtctgcaccaacaccgccggctccttctcctgcagggactgtgaggagggttaccggcccagccccctgggccacacctgcgaagat

LTBP2 G V C T N T A G S F S C R D C E E G Y R P S P L G H T C E D

3961: gtggatgagtgtaaggacttccagagcagctgcctgggaggcgagtgcaagaacacggctggctcctatcagtgcctctgtcccacaggc

LTBP2 V D E C K D F Q S S C L G G E C K N T A G S Y Q C L C P T G

4051: ttccagctggccaatggcaccacgtgtgaggatgtggacgagtgcgtgggagaggagtactgtgcaccccgcggcgagtgcctcaacagc

LTBP2 F Q L A N G T T C E D V D E C V G E E Y C A P R G E C L N S

4141: cacgggtccttcttctgtctctgcgcaccgggcttcgccagcgccgaggggggcaccagctgccaggatgtggacgaatgtgcagtcaca

LTBP2 H G S F F C L C A P G F A S A E G G T S C Q D V D E C A V T

4231: gaccggtgtctgggaggacactgtgtcaacaccgagggctccttcaactgtctgtgtgaaaccggcttccagccctccccagagagcggg

LTBP2 D R C L G G H C V N T E G S F N C L C E T G F Q P S P E S G

4321: gagtgtgtggatattgacgagtgtaaggaccacggcgactccgtatgtggggcctggaggtgtgagaacagccctggctcctaccgctgt

LTBP2 E C V D I D E C K D H G D S V C G A W R C E N S P G S Y R C

4411: gtcctagcctgccagcctggcttccacatggctccgactggagactgcattgacatagacgagtgtgccaacgacaccatgtgtgggagc

LTBP2 V L A C Q P G F H M A P T G D C I D I D E C A N D T M C G S

4501: cacggcttctgcgacaacactgacggctccttccgctgcctctgtgaccagggcttcgagacttcgtcctccggctgggaatgtgttgac

LTBP2 H G F C D N T D G S F R C L C D Q G F E T S S S G W E C V D

4591: gtgaacgagtgtgagctcatgctggcggtgtgtggggcggcgctctgtgagaatgtggagggctccttcctgtgcctctgtgccagtgac

LTBP2 V N E C E L M L A V C G A A L C E N V E G S F L C L C A S D

4681: ctggaggagtacgacgctcaggaggggcactgccgcccgcgggtggctggaggtcagagtattcctgaggccccgccaggggaccacccc

LTBP2 L E E Y D A Q E G H C R P R V A G G Q S I P E A P P G D H P

4771: ccgggccccatccgcatggagtgctactctgggcacaatgaccagctgccctgctccagccttctgggccggaacaccacacaggccgag

LTBP2 P G P I R M E C Y S G H N D Q L P C S S L L G R N T T Q A E

4861: tgctgctgcactcagggtgccagctggggagacgcctgtgacctctgcccagctgaggactcagtggaattcagcgagatctgccctagt

LTBP2 C C C T Q G A S W G D A C D L C P A E D S V E F S E I C P S

4951: ggtaaaggctacatccctgtggacggagcctggatgtttggacagaccacgtacacagatgcggacgagtgtgtgatgttcgggcctggg

LTBP2 G K G Y I P V D G A W M F G Q T T Y T D A D E C V M F G P G

5041: ctctgccagaacggccggtgcctcaacacggtgcccggctacgtctgcctgtgccatcccggctaccactacaacgccgcccacaggaag

LTBP2 L C Q N G R C L N T V P G Y V C L C H P G Y H Y N A A H R K

5131: tgtgaggatcatgacgagtgccaggacatggtctgcgagaacggtgagtgtgtgaacactgaaggctctttccactgcttctgcagcccc

LTBP2 C E D H D E C Q D M V C E N G E C V N T E G S F H C F C S P

5221: ccgctcaccctggacctcggccagcagcgctgcgtgaacagcaccggcggcacggaggacctgcctgaccacgacatccacatggacatc

LTBP2 P L T L D L G Q Q R C V N S T G G T E D L P D H D I H M D I

5311: tgctggaaaagagtcaccaattatgtgtgcagccaacccctgcacgggcgccgcaccacctacacagaatgctgctgtcaggatggcgag

LTBP2 C W K R V T N Y V C S Q P L H G R R T T Y T E C C C Q D G E

5401: gcctggagccagcagtgtgccctgtgcccccccaggagctctgaggtctacgctcagctgtgcaacgtggcccggatcgaagccgagcag

LTBP2 A W S Q Q C A L C P P R S S E V Y A Q L C N V A R I E A E Q

5491: gaggctggagtccacttccggccaggctatgagtacggccccgggcccgaggacctgcattacagcctctacggcccagacggggccccc

LTBP2 E A G V H F R P G Y E Y G P G P E D L H Y S L Y G P D G A P

5581: ttctacaactacctgggtcccgaggacaccgtcccagagccacccttccccaacacagccagccgcccaggggaccacctaccagttctt

LTBP2 F Y N Y L G P E D T V P E P P F P N T A S R P G D H L P V L

5671: gagccccccctgcagccctcagaactccagccccactacgtggccagccacccagagcaccaggccggcttcgaagggcttcaggcagag

LTBP2 E P P L Q P S E L Q P H Y V A S H P E H Q A G F E G L Q A E

5761: gaatgcggcatcctgaacggctgtgagaacggccgctgtgtgcgagtgagggagggctacacctgtgactgctttgagggcttccagctg

LTBP2 E C G I L N G C E N G R C V R V R E G Y T C D C F E G F Q L

5851: gacatgacccacatggcctgcgtggatatcaatgagtgtgacgacttgaacgggcctgctgcgctctgtgcccacggtcactgcgagaac

LTBP2 D M T H M A C V D I N E C D D L N G P A A L C A H G H C E N

5941: accgagggctcctaccgctgccattgctccccaggttatgtggccgaggccggccccccacactgtacctccaaggaatagcagtgaggg

LTBP2 T E G S Y R C H C S P G Y V A E A G P P H C T S K E STOP

6031: ctcagtgtgggcagctacctggaaatgggcccaggccacacaggcaggggcctcgagaaggctttcctagctgggaagacaccgtgaaga

6121: agcaggcagaaggccctgcagcccagctcccagccagccccgcctgcttttcatctctcccagcttaggctctggctgagcttctgtcac

6211: tgccttcatgctgccgtgcccttgcttggctcaaacaccaccaaatgctttaatgcttcagccactggccatgaggaccagtctgccatt

6301: tgtcctggccttgctatggatgcttcctaaaggatggccctcatccaccccctcaagctgtgcaaacacgcaaggtcactcggactcaca

6391: ttgcagataccctttcccagccatgatccacaggacatcctgatgattccacaactgggtcagaggtcacatctgcccagggatggtcct

6481: tcagtatctcttgagcaaaaaaggattgggggggggggggggttggggagtgactccaaggccgcctccagagaaccaacacttcactca

6571: gccaatccggtctgggccagattttgccacagctccatccaatagcagttctgtggccccagggaggagcagatcagctcagctcatctc

6661: ggaagagcaaaaactgataccagcttgctgagtgtagagacacgagtgaaggggaacaagaagcctaagaaagcagcaagagggcaatat

6751: gaaaaatacggggagttgttaagaaagggggagccaaggctttcaacaagtctaaagaaaacgttcagtaacttcgggtaaacactcacc

6841: atctacccatgataattcgcgtgcacccagcaaaggctgccgcaggacagggtgcccatctccgaagacctgtattaaatacatactgct

6931: tcttacaggaaacgaatctctcctgggttcccttttgtggatgccagttttgaggtactaaaaataaaggggcctattttctagcttgtg

7021: agatacagtgtagtcttatttaggggaagccagatttcctatgttgtttctgtattctcagatgcccctgccatctttctttcaaaaatg

7111: ggatggctgctatccctctcagtttacaagtagagacccactccaaccttggccagccaggagtttagaaccattgtggaataaaaagat

7201: gtacatctgggcttttctgtttttgtttttatttcatattaaaccaaatttctttaccatgttggctaagtctaattattagacatgagg

7291: ctgtgcctaccctcttgccaactctgccaatagcctaggattgcgatccaatgggaaaaggctccttcttcttctgagacaaggaaagct

7381: ctgcctttgcatttgtctgatgaaaggcaatgtaaatgaacgagactccatgcgcctgaagacatagggacgaatgtcattttcttaatt

7471: taatctgtcctgtcctagcggttgctctgattgtcagccatcccataataacttccaaatgcctgttatgtgccaggcactgtgttgagt

7561: gctggacacaaatttcatttaatcatccaacaatcctactgactatttttattcccaacttacagctgaggaaattgagacacacaagag

7651: tttaaacaatttgccctaggtcactctactagctaagatgttttcagccaacaaaactatatagttcttacacatgtgaaaatattctga

7741: ccttctttcgtaataaaagaaatgtgaattaaaaccacactgagatactttttaaaaattgtcagattggcaaggatcaaaacgtttaag

7831: actcctagtctgcaagcatctgatgaaagaggcactcacatacacagctaacagggacggaaactgatagaacttctgaaaaggataata

7921: tggcaacgtctttcaaacttataaaagctcttagtctttgaatcttctaggaatttaaccttcagatatttctcaccaaatgtgaaacga

8011: cacacgtcagaggttatttattatagcattacttgtaacagtaaaagattagaaactattagttgaaacggaggcctaaaaaaggaggaa

8101: aaaaaaaaaagtggctgtaaatgccttgataagaaatccaaaccagacgaaggggggaaagaaggtcacataacaaagtgtaatatatgc

8101: tgctatttatctaaagaaagggggaagacagctgggtatctgctgacctcaccctcgccactctccttacgccccacggttcagagaact

8191: tcttaccactcttcagtacacctgctggcactgtgcctcctctcaaccctcgccttcctgctcacccttccagaaaaacttcctgcacat

8281: acacggatttcttcgtatcaaaacacaaagaccactctatgcatgctgttctgcactttgcttttttcacttaatccgtattttcacata

8371: aagagttgtctcgttctatattgtaattgtatgctattccactgaatggatcaatcatagtttgtaatcagtcccctattgacagatgtt

8461: tccattgtttcctatctcttgctatttataagtaatgctgtaatgaacattcttgtatgttatttcacattatatctatggatcatacct

8551: gtagggtaaattcttagaaacaacagtggtttgtggggagggtgacagggtacatgggtacaggattgggacttttcactgtatcccctt

8641: ttgtgtctttttgaattttgtaccgtgtgtattaccttttcaaaaaaataaaatatttttaaatacagctgctggccacagaccacttaa

8731: atgctccctcctttatgtctttatctgacaattaaagccccgcaattattgcaaaacttcttaaagcaaacttcctttaaacagccctgc

8821: agcatttctgtggagaaatggaaaacagggattctgaaagagcaaccataaacaccaatgctagatacccagaaagtagaggagtttcct

8911: gcctttgagacacacatgcaaaaccgtaattaatgttgcactggacagtgtcaggtcccgtccactaccagcggcacgagacccactcct

9001: ggccagcactgacgggagggctacgccagatgatcggtgccgcccacttctcaatacaggatgctcctaccacatgtcagccctccgttt

9091: gaggctttgtaacagaagaccgaaagaacgggtttcaattcttgagaagttagaaagttcttggacacctacttctaacccctttacttc

9181: taaacctgttttctcctaatcccttgtaatcagtcaacattcccacacaaatgaagggtgggtgggacacctacagtgtacataccacca

9271: gtcactatcagtcatggaactgaagcgtatgattccacaaggtaagatctggatgttgacaccgtagagcagattcacaacatgaagcag

9361: acgcggggctcccttgtctttggttcttgctaaagcaaaggggggacatgccgccccagatactctctaactttgcataaataaaaccaa

9451: ttctgcaacaaatagggcctttccccttgaaagtaatggggaagcaaaaaaaaaaaaaaagtctcccaatggatactagaggagtgtcaa

9541: gttttcataaatgaatttgcacagtgagcaaaatttgtgaaaacacctgctggtctttactgcttgccagaaagagggcatctggtcaaa

9631: aagggcctgcaaaagaccctaaacccattttactatacttaactatttgttccaagccaactgcccaccgtcccctcgatgctatcattt

9721: ctctatgccccgtgcttttaaaatttctacgtcatcctactccaatggaggcttcttgttatcacttcagattttcccctgaggctttct

9811: cagtgtaataaagtgaggtgatttctcccaaacccaccctttctaaaacacagaagtccaaatctcccattaacagcaagtacttccata

9901: aaggcattcaaagattcggttcctgtctgactactgcaatttaatgatttatttagcagctaaagctaccttgcatgtgctcggcatgag

9991: ttttaaaatcaccaaatcatggagcacctggctggctcagtcagtgtaacatgcaactcttgatctcagggtcatgagttcaaggcccat

10081: gttgggtacagggcttccttcaaaaaataaacaaaaataaagtgcactctttaaacaggttctaatacataaaatgtagcaaaatctctt

10171: tttaacgaggatgaaaaaacttagtctgagctgaagatcttatcaggcccaggtactgatgcatctttaggtatctaagtaaagccacac

10261: agatgcccctatggtatgctgctctctgcctccttccatggaaaactgccaagaaagctcaggtatctgagcctccctttgtttcctctt

10351: tgtactcacaataggcttgctttctccaaattctaggttttcccacattgaaaatgtaagtacctaagcttctgtcccagatacttttct

10441: ctaaactctgcatttccctttttaatggtaaattcttttaatgaatgaccttaattccaatccagtccagaaatgcaagttttgccgtaa

10531: caaactttaaggctcttgaggaaaacaagggtttaaccagcaattcttccatcatcaatttggttgtggttttccccgacatgagcatta

10621: ataaatcatttgttatggtagtggtatttctttttttagttgtccatacagttccagctcccccagccctgtcacacacatgccacaact

10711: atttctagatctgtagactaattaagtgccatacccaaacaagtactttaaaattcatcggaattttggcttaaaaacctttccttgggg

10801: cgcctgggtggctcattcgttccgttaagcaccagactcttgatttcagctcaggtcatgatctcatggttcatgagatgcagctgtcag

10891: cacagagcctgcttgggattctctctctccccctctctcccctcccaactcacactctctctctcaaaataaataaataaaacttaaaaa

10981: aaaaagttttcttcagtgaccctaaaataaagctgccaggtttaacaaacagaaatacacaatgtccctgacgcctgggtggctcagtcg

11071: attaagcatccaactcttggttttggcttaggtcatgatctcacagtttgtgagttcgagccccacactgggctccgcaatgtgtgtggc

11161: gcctccttgaaattctctcttcctctctctctctgcccctccccctcccccactcgctgtctctctcaaaataaataaacttaaaaaaaa

10251: aaaaaaccacagtgcccagttaaatttgaatttcaaataaaccacacataatttttagtataaatatgtttcacgaaatatttggggcac

11341: acttatactaaaaattacatgttatttgaaattctaatttaactgggcatcttacattttatctggtaatccctaactatccatatccat

11431: accaaca
