# Supplementary material for: A Mutation in LTBP2 Causes Congenital Glaucoma in Domestic Cats (Felis catus)
Source: PLoS One. 2016 May 5;11(5):e0154412. doi: 10.1371/journal.pone.0154412 (PMC4858209; doi:10.1371/journal.pone.0154412)
Supplement: S2 Fig — Sequence homology to human LTBP2 is slightly lower in mice (77% over 98% length). (DOCX) [file pone.0154412.s002.docx]

Cat to human (NM_000428.2) 87% identity over 100% length

Cat to mouse (NP_038617.3) 77% over 98% (mouse is shorter)

CLUSTAL O(1.2.1) multiple sequence alignment

Mus MESTSPRGLRCPQLCSHSGAMRAPTTARCSGCIRRVRWRGFLPLVLAVLMGTSHAQRDSI

Felis --------------------MRPPTTHRCTGRALRNPCGSLLALTLALFVGMGHAQRDPV

Homo --------------------MRPRTKARSPGRALRNPWRGFLPLTLALFVGAGHAQRDPV

** *. *. * * .:* *.**:::* .***** :

Mus GRYEPASRDANRLWHPVGSHPAAAAAKVYSLFREPDAPVPGLSPSEWNQPAQGNPGRLAE

Felis ERSEPAGREANRLRRPGGSHPAAAAAKVYSLFREQDAPVPGLPPTERAQLGWGSARRPAN

Homo GRYEPAGGDANRLRRPGGSYPAAAAAKVYSLFREQDAPVAGLQPVERAQPGWGSPRRPTE

* ***. :**** :* **:************** **** ** * * * . *. * ::

Mus AEARRPPRTQQLRRVQPPVQTRRSHPRGQQQIA--ARAAPSVARLETPQRPAA----ARR

Felis ALARRPPRAQQPRRAQPPAQTWRSSPLGQQQPAVRARAAPALPRLGTPQRPGAAPPTPPR

Homo AEARRPSRAQQSRRVQPPAQTRRSTPLGQQQPAPRTRAAPALPRLGTPQRSGAAPPTPPR

* **** *:** **.***.** ** * **** * :****:: ** **** .* *

Mus GRLTGRNVCGGQCCPGWTTSNSTNHCIKPVCQPPCQNRGSCSRPQVCICRSGFRGARCEE

Felis GRLTGRNVCGGQCCPGWTTANSTNHCIKPVCQPPCQNRGSCSRPQLCVCRSGFRGARCEE

Homo GRLTGRNVCGGQCCPGWTTANSTNHCIKPVCEPPCQNRGSCSRPQLCVCRSGFRGARCEE

*******************:***********:*************:*:************

Mus VIPEEEFDPQNARPVPRRSVERAPGPHRSSEARGSLV-TRIQPLVP----PPSPPPS---

Felis VIPEEEFDPQNSRPAPRRSAEGSPNPRGSSAARESTTTPRTRSLASQLPPARSLPPARTL

Homo VIPDEEFDPQNSRLAPRRWAERSPNLRRSSAAGEGTL-ARAQPPAPQSPPAPQSPPAGTL

***:*******:* .*** .* :* : ** * . * : . . **:

Mus RRLSQPWPLQQHSGPSRTVRRYPATGANGQLMSNALPSG--LELRDSSPQAAHVNHLSPP

Felis SGLSQTQPSQQHVGLSRTARLYPTTAASGQLTSNALPAGPGLERRDGTQQAAYLDHPSSP

Homo SGLSQTHPSQQHVGLSRTVRLHPTATASSQLSSNALPPGPGLEQRDGTQQAVPLEHPSSP

*** * *** * ***.* :*:: *..** ***** * ** **.: **. ::* * *

Mus WGLNLTEKIKKIKVVFTPTICKQTCARGRCANSCEKGDTTTLYSQGGHGHDPKSGFRIYF

Felis WGLNLTEKIKKIKIVFTPTICKQTCARGHCSNSCERGDTTTLYSQGGHGHDPKSGFRIYF

Homo WGLNLTEKIKKIKIVFTPTICKQTCARGHCANSCERGDTTTLYSQGGHGHDPKSGFRIYF

*************:**************:*:****:************************

Mus CQIPCLNGGRCIGRDECWCPANSTGKFCHLPVPQPDREPAGRGSRHRTLLEGPLKQSTFT

Felis CQIPCLNGGRCIGRDECWCPTNSTGKFCHLPAPNLDKGPPERGSRHRALLEVPLRQSTFT

Homo CQIPCLNGGRCIGRDECWCPANSTGKFCHLPIPQPDREPPGRGSRPRALLEAPLKQSTFT

********************:********** *: *: * **** *:*** **:*****

Mus LPLSNQLASVNPSLVKVQIHHPPEASVQIHQVARVRGELDPVLEDNSVETRASRRPHGNL

Felis LPLSNQLASVNPSLVKVHIHHPPEASVQVHQVARVRGEA---PEENSVETRPSPRLPASP

Homo LPLSNQLASVNPSLVKVHIHHPPEASVQIHQVAQVRGGVEEALVENSVETRPPPWLPASP

*****************:**********:****:*** :****** ..

Mus GHSPWASNSIPARAGEAPRPPP-VLSRHYGLLGQCYLSTVNGQCANPLGELTSQEDCCGS

Felis RHSHWDSNSIPARSGEAPQPPPPAAPRPPGLLGRCYLSSVNGQCANPLLELTAQEDCCGS

Homo GHSLWDSNNIPARSGEPPRPLPPAAPRPRGLLGRCYLNTVNGQCANPLLELTTQEDCCGS

** * **.****:** *:* * . * ****:***.:********* ***:*******

Mus VGTFWGVTSCAPCPPRPAFPVIENGQLECPQGYKRLNLSHCQDINECLTLGLCKDSECVN

Felis VGAFWGVTSCAPCPPRPASPVVENGQLECPQGYKRLNLTHCQDINECLTLGLCKDSECVN

Homo VGAFWGVTLCAPCPPRPASPVIENGQLECPQGYKRLNLTHCQDINECLTLGLCKDAECVN

**:***** ********* **:****************:****************:****

Mus TRGSYLCTCRPGLMLDPSRSRCVSDKAVSMQQGLCYRSLGSGTCTLPLVHRITKQICCCS

Felis TRGSYLCTCRPGLMLDPSRSRCVSDKAVSMQQGLCYRSLGAGTCTLPLAQSITKQICCCS

Homo TRGSYLCTCRPGLMLDPSRSRCVSDKAISMLQGLCYRSLGPGTCTLPLAQRITKQICCCS

***************************:** ********* *******.: *********

Mus RVGKAWGSTCEQCPLPGTEAFREICPAGHGYTYSSSDIRLSMRKAEEEELASPLREQTEQ

Felis RVGKAWGSKCERCPLPGTEGFREICPAGHGYTYSSSHIRLAMRKAEEEELARPSREQAQK

Homo RVGKAWGSECEKCPLPGTEAFREICPAGHGYTYASSDIRLSMRKAEEEELARPPREQGQR

******** **:*******.*************:**.***:********** * *** ::

Mus STAPPPGQAERQPLRAATATWIEAETLPDKGDSRAVQITTSAPHLPARVPGDATGRPAPS

Felis SYGTLLGPAERQPLRAVTDTWLEAETIPDKGDSQAGQVTTSVTQVPAWVPGNATERPTPP

Homo SSGALPGPAERQPLRVVTDTWLEAGTIPDKGDSQAGQVTTSVTHAPAWVTGNATTPP---

* . * *******..* **:** *:******:* *:***. : ** * *:** *

Mus LPGQGIPESPAEEQVIPSSDVLVTHSPPDFDPCFAGASNICGPGTCVSLPNGYRCVCSPG

Felis LPGQEIPDNPEEERVTTPHDGLEARGPSGIDRCATGATNICGPGTCVTLPDGYKCLCSPG

Homo MPEQGIAEI-QEEQVTPSTDVLVTLSTPGIDRCAAGATNVCGPGTCVNLPDGYRCVCSPG

:* * * : **:* * * : . :* * :**:*:*******.**:**:*:****

Mus YQLHPSQDYCT-------------------------------------------DIDECE

Felis YQLHPSQAYCTDDNECLRDPCAGRGRCVNRVGSYSCFCYPGYTLATSGTTQECQDIDECE

Homo YQLHPSQAYCTDDNECLRDPCKGKGRCINRVGSYSCFCYPGYTLATSGATQECQDINECE

******* *** **:***

Mus QPGVCSGGRCSNTEGSYHCECDRGYIMVRKGHCQDINECRHPGTCPDGRCVNSPGSYTCL

Felis QPGVCSGGQCTNTEGSYDCQCDQGYIMVRKGHCQDINECRHPGTCPDGRCVNSPGSYTCL

Homo QPGVCSGGQCTNTEGSYHCECDQGYIMVRKGHCQDINECRHPGTCPDGRCVNSPGSYTCL

********:*:******.*:**:*************************************

Mus ACEEGYVGQSGSCVDVNECLTPGICTHGRCINMEGSFRCSCEPGYEVTPDKKGCRDVDEC

Felis ACEEGYRGQSGSCVDVNECLTPGVCAHGKCINLEGSFRCSCEPGYEVTSDEKGCQDVDEC

Homo ACEEGYRGQSGSCVDVNECLTPGVCAHGKCTNLEGSFRCSCEQGYEVTSDEKGCQDVDEC

****** ****************:*:**:* *:********* ***** *:***:*****

Mus ASRASCPTGLCLNTEGSFTCSACQSGYWVNEDGTACEDLDECAFPGVCPTGVCTNTVGSF

Felis ASRASCPTGLCLNTEGSFTCSACESGYWVNEDGTACEDLDECAFPGVCPSGVCTNTAGSF

Homo ASRASCPTGLCLNTEGSFACSACENGYWVNEDGTACEDLDECAFPGVCPSGVCTNTAGSF

******************:****:.************************:******.***

Mus SCKDCDRGYRPNPLGNRCEDVDECEGPQSSCRGGECKNTEGSYQCLCHQGFQLVNGTMCE

Felis SCRDCEEGYRPSPLGHTCEDVDECKDFQSSCLGGECKNTAGSYQCLCPTGFQLANGTTCE

Homo SCKDCDGGYRPSPLGDSCEDVDECEDPQSSCLGGECKNTVGSYQCLCPQGFQLANGTVCE

**:**: ****.***. *******: **** ******* ******* ****.*** **

Mus DVNECVGEEHCAPHGECLNSLGSFFCLCAPGFASAEGGTRCQDVDECAATDPCPGGHCVN

Felis DVDECVGEEYCAPRGECLNSHGSFFCLCAPGFASAEGGTSCQDVDECAVTDRCLGGHCVN

Homo DVNECMGEEHCAPHGECLNSHGSFFCLCAPGFVSAEGGTSCQDVDECATTDPCVGGHCVN

**:**:***:***:****** ***********.****** ********.** * ******

Mus TEGSFSCLCETGFQPSPDSGECLDIDECEDREDPVCGAWRCENSPGSYRCILDCQPGFYV

Felis TEGSFNCLCETGFQPSPESGECVDIDECKDHGDSVCGAWRCENSPGSYRCVLACQPGFHM

Homo TEGSFNCLCETGFQPSPESGECVDIDECEDYGDPVCGTWKCENSPGSYRCVLGCQPGFHM

*****.***********:****:*****:* * ***:*:**********:* *****::

Mus APNGDCIDIDECANDTVCGNHGFCDNTDGSFRCLCDQGFETSPSGWECVDVNECELMMAV

Felis APTGDCIDIDECANDTMCGSHGFCDNTDGSFRCLCDQGFETSSSGWECVDVNECELMLAV

Homo APNGDCIDIDECANDTMCGSHGFCDNTDGSFRCLCDQGFEISPSGWDCVDVNECELMLAV

**.*************:**.******************** * ***:**********:**

Mus CGDALCENVEGSFLCLCASDLEEYDAEEGHCRPRVAGAQRIPEVRTEDQAPSLIRMECYS

Felis CGAALCENVEGSFLCLCASDLEEYDAQEGHCRPRVAGGQSIPEAPPGDHPPGPIRMECYS

Homo CGAALCENVEGSFLCLCASDLEEYDAQEGHCRPRGAGGQSMSEAPTGDHAPAPTRMDCYS

** ***********************:******* **.* : *. *: *. **:***

Mus EHNGGPPCSQILGQNSTQAECCCTQGARWGKACAPCPSEDSVEFSQLCPSGQGYIPVEGA

Felis GHNDQLPCSSLLGRNTTQAECCCTQGASWGDACDLCPAEDSVEFSEICPSGKGYIPVDGA

Homo GQKGHAPCSSVLGRNTTQAECCCTQGASWGDACDLCPSEDSAEFSEICPSGKGYIPVEGA

:: ***.:**:*:*********** **.** **:***.***::****:*****:**

Mus WTFGQTMYTDADECVLFGPALCQNGRCLNIVPGYICLCNPGYHYDASSRKCQDHNECQDL

Felis WMFGQTTYTDADECVMFGPGLCQNGRCLNTVPGYVCLCHPGYHYNAAHRKCEDHDECQDM

Homo WTFGQTMYTDADECVIFGPGLCPNGRCLNTVPGYVCLCNPGFHYDASHKKCEDHDECQDL

* **** ********:***.** ****** ****:***.**:**:*: :**:**:****:

Mus ACENGECVNTEGSFHCLCNPPLTLDLSGQRCVNSTSSTEDFPDHDIHMDICWKKVTNDVC

Felis VCENGECVNTEGSFHCFCSPPLTLDLGQQRCVNSTGGTEDLPDHDIHMDICWKRVTNYVC

Homo ACENGECVNTEGSFHCFCSPPLTLDLSQQRCMNSTSSTEDLPDHDIHMDICWKKVTNDVC

.***************:*.*******. ***:***..***:************:*** **

Mus SQPLRGHHTTYTECCCQDGEAWSQQCALCPPRSSEVYAQLCNVARIEAERGAGIHFRPGY

Felis SQPLHGRRTTYTECCCQDGEAWSQQCALCPPRSSEVYAQLCNVARIEAEQEAGVHFRPGY

Homo SEPLRGHRTTYTECCCQDGEAWSQQCALCPPRSSEVYAQLCNVARIEAEREAGVHFRPGY

*:**:*::*****************************************: **:******

Mus EYGPGLDDLPENLYGPDGAPFYNYLGPEDTAPEPPFSNPASQPGDNTPVLEPPLQPSELQ

Felis EYGPGPEDLHYSLYGPDGAPFYNYLGPEDTVPEPPFPNTASRPGDHLPVLEPPLQPSELQ

Homo EYGPGPDDLHYSIYGPDGAPFYNYLGPEDTVPEPAFPNTAGHSADRTPILESPLQPSELQ

***** :** .:*****************.*** * * *.: .*. *:** ********

Mus PHYLASHSEPLASFEGLQAEECGILNGCENGRCVRVREGYTCDCFEGFQLDAAHMACVDV

Felis PHYVASHPEHQAGFEGLQAEECGILNGCENGRCVRVREGYTCDCFEGFQLDMTHMACVDI

Homo PHYVASHPEPPAGFEGLQAEECGILNGCENGRCVRVREGYTCDCFEGFQLDAAHMACVDV

***:*** * *.************************************** :******:

Mus NECEDLNGPAALCAHGHCENTEGSYRCHCSPGYVAEPGPPHCAAKE

Felis NECDDLNGPAALCAHGHCENTEGSYRCHCSPGYVAEAGPPHCTSKE

Homo NECDDLNGPAVLCVHGYCENTEGSYRCHCSPGYVAEAGPPHCTAKE

***:******.**.**:******************* *****::**
